# Comprehensive pan-cancer analysis of TRAP1 and its experimental validation in hepatocellular carcinoma

**DOI:** 10.1007/s12672-025-04238-9

**Published:** 2025-12-20

**Authors:** Shuaikun Shan, Tiantian Wang, Fangzheng Sun, Ludong Zhao, Shuang Liang, Zhenghua Wang

**Affiliations:** 1 First Affiliated Hospital of Jinzhou Medical University, Jinzhou, China; 2 Present Address: Xinxiang Central Hospital, Xinxiang, Henan China; 3 Jinzhou Medical University, 121001 Jinzhou, People’s Republic of China; 4 Emergency Rescue Center of Binhai New Area, Tianjin, China; 5 Department of Oncology, First Affiliated Hospital of Jinzhou Medical University, Jinzhou Medical University, Jinzhou, Liaoning China

**Keywords:** Tumor necrosis factor receptor-associated protein 1, Cancer, Prognosis, Immune infiltration, Bioinformatics

## Abstract

**Purpose:**

Tumor necrosis factor receptor-associated protein 1 (TRAP1) is essential for carcinogenesis and the advancement of cancer, making it a promising therapeutic target in oncology. Nevertheless, comprehensive bioinformatic analyses of TRAP1 across diverse cancer types are limited. Herein, we analyzed TRAP1 across all cancer types, focusing on its expression in relation to prognosis, immune infiltration, and the mammalian target of rapamycin and receptor tyrosine kinase signaling pathways.

**Methods:**

We evaluated TRAP1’s clinical relevance for prognostic predictions and its association with tumor immunity and metabolism. TRAP1’s function in hepatocellular carcinoma cell invasion, migration, and proliferation was examined in vitro using wound healing assays and the cell counting kit-8; apoptosis was examined through reactive oxygen species detection.

**Results:**

We found that TRAP1 significantly predicts cancer prognosis and is closely linked to immune and metabolic tumor characteristics. In liver cancer cells, TRAP1 knockdown prevented invasion, migration, and proliferation; increased reactive oxygen species; and promoted apoptosis.

**Conclusion:**

In summary, this study reveals the critical clinical significance of TRAP1 across multiple cancer types through a pan-cancer analysis. Further in vitro experiments demonstrate that knocking down TRAP1 significantly suppresses malignant phenotypes of tumor cells in hepatocellular carcinoma by inducing oxidative stress and apoptosis. Thus, TRAP1, particularly in liver cancer, represents a highly promising prognostic biomarker and a novel metabolic therapeutic target. These findings provide direction for subsequent research on TRAP1 and strongly support its potential for translational exploration in hepatocellular carcinoma treatment.

**Supplementary Information:**

The online version contains supplementary material available at 10.1007/s12672-025-04238-9.

## Introduction

Recent advancements in tumor genomic technologies have ushered in a new era of cancer research characterized by pan-cancer analyses. This approach leverages genomic techniques to explore various tumor types, revealing shared and distinct patterns of genomic and cellular alterations [1]. Such insights are crucial for understanding tumor development and identifying possible therapeutic strategies across various malignancies.

Tumor necrosis factor receptor-associated protein 1 (TRAP1), a member of the Hsp90 chaperone family, is mostly found in the mitochondrial matrix [2]. Cellular homeostasis and mitochondrial integrity depend on TRAP1, and recent research highlights TRAP1 dysregulation as a key factor in the progression and metastasis of various cancers. As a regulatory protein, TRAP1 coordinates metabolic reprogramming between aerobic glycolysis and oxidative phosphorylation (OXPHOS) in cancer via modifying carcinogenic proteins and signaling networks [3, 4]. Additionally, TRAP1 modulates the cell cycle to promote the growth of new cells [5], reduces reactive oxygen species (ROS) production to protect against apoptosis, enhances resistance to chemotherapy [6], and stimulates mitochondrial fission, which aids in the spread of tumors (Purushottam Dharaskar et al. 2020).

Aberrant TRAP1 expression has been connected to the emergence of several malignancies, including glioblastoma (GBM) [7], nasopharyngeal carcinoma [8], colorectal adenocarcinoma (COADREAD) [9], prostate cancer (PRAD) [10], stomach adenocarcinoma (STAD) [11], esophageal carcinoma (ESCA), renal cell carcinoma (RCC) [12], and non-small cell lung cancer [13]. Notably, TRAP1 may have tumor-suppressive properties in some malignancies, including cervical cancer, bladder cancer, and clear cell RCC [14]. A review of previous studies suggests that this dual role may depend on the specific metabolic characteristics of each tumor type [15]. Given its central role in tumorigenesis, selective targeting of TRAP1 presents a promising strategy for developing novel anticancer therapies,however, comprehensive bioinformatic analyses of TRAP1 across diverse cancer types are limited. This study examines TRAP1 expression and its prognostic significance in various cancers using multiple databases. Subsequently, we selected hepatocellular carcinoma (HCC as a representative model for in-depth in vitro functional validation to assess its potential as a therapeutic target. Online Resource 1 lists all cancer types and their abbreviations.

## Methods

### TRAP1 expression levels and subcellular localization

Data from the Human Protein Atlas (HPA; https://www.proteinatlas.org/) and Genotype-Tissue Expression (GTEx; https://gtexportal.org/) were used to assess TRAP1 mRNA expression in normal tissues (N = 13,084). TRAP1's subcellular localization was evaluated through immunofluorescence staining in three human cell lines from the HPA. The UCSC Xena database (https://xenabrowser.net/) provided uniformly processed RNA-Seq expression data (in TPM units, with batch correction). This database integrates datasets from The Cancer Genome Atlas (TCGA) and GTEx, resulting in a dataset of 34 cancer types after excluding those with < 3 samples. The SangerBox web program (version: 3.0) (http://vip.sangerbox.com/) analyzed the differences in TRAP1 expression between cancerous and healthy tissues. Expression data were transformed using log2(x + 0.001) to conform to a normal distribution. Differential expression across tumors was determined using R software (version 3.6.4). Group comparisons were performed using the unpaired Wilcoxon rank-sum test, with multiple testing correction applied via the Benjamini-Hochberg (BH) false discovery rate (FDR) method. An FDR < 0.05 was set as the threshold for statistical significance. Through the UALCAN portal (http://ualcan.path.uab.edu/), under “Proteomics,” the Clinical Proteomic Tumor Analysis Consortium dataset was used to further investigate the expression of the TRAP1 protein in both normal and malignant tissues. TRAP1 expression was investigated across cancer stages using the GEPIA tool (version: 2.0) (http://gepia2.cancer-pku.cn/#analysis).

### Analysis of survival prognosis

Using TCGA clinical data, the optimal cutoff value for ENSG00000126602 (TRAP1) was calculated using the R package maxstat (maximally selected rank statistics with several p-value approximations, version: 0.7–25). Based on this value, patients were stratified into high- and low-expression groups. Prognostic differences between the two groups were subsequently analyzed using the survfit function from the R package survival (version: 3.5.7) to fit survival curves, and survival disparities were assessed using the log-rank test.

### DNA mutation analysis

The cBioPortal database (https://www.cbioportal.org/) provided the TRAP1 mutation data. Using the “TCGA PanCancer Atlas Studies,” we input “TRAP1″ into the “Cancer Types Summary” module to examine the mutation landscape, including mutation types, copy number alterations, and mutation frequency. Detailed mutation site information was retrieved from the “Mutations” module.

### DNA methylation analysis

By choosing the "TCGA" dataset and entering "TRAP1," the UALCAN database provided the DNA methylation information for the TRAP1 gene in both normal and cancerous tissues.

### TRAP1 expression and its correlation with immune checkpoints and cell infiltration

We used the SangerBox platform (version: 3.0) to examine the relationship between immunological checkpoints (ICPs) and TRAP1 expression. TRAP1 and 60 immune checkpoint pathway genes—24 inhibitory and 36 stimulatory—were expressed in tumor tissues. The log2(x + 0.001) technique was used to transform each expression value, and Pearson correlations between TRAP1 and the immunological marker genes were computed. We analyzed the relationship between immune infiltration scores and TRAP1 expression. Data on TRAP1 expression were gathered for various tumor types, and stroma, immune, and ESTIMATE scores were computed for every patient using the ESTIMATE R package (version 1.0.13), providing each patient’s immune infiltration scores. Subsequently, the cor.test function from the psych R package (version 2.1.6) was used to analyze significant associations between TRAP1 expression and immune infiltration scores, with the Benjamini-Hochberg (BH) method similarly applied for FDR correction. An FDR < 0.05 was defined as statistically significant for correlations.

### TRAP1-related gene and protein enrichment analysis

We used the STRING database (https://cn.string-db.org/) to analyze the top 50 proteins interacting with TRAP1 in order to study its network of protein–protein interactions. We selected Homo sapiens as the organism, entered “TRAP1” in the “Multiple proteins” area, and obtained information on interacting proteins. In GEPIA2’s “Similar Genes Detection” module, we determined the top 100 TRAP1-related genes, selecting five for “Correlation Analysis” to generate scatter plots illustrating their relationships. Furthermore, we generated heatmaps for these five genes in TCGA cancer data using TIMER2.0 (http://timer.comp-genomics.org/timer/) in the “Exploration” part of the “Gene_Corr” module. Subsequently, enrichment analyses were performed using the DAVID database (https://david.ncifcrf.gov/) and Kyoto Encyclopedia of Genes and Genomes (KEGG), selecting the official gene symbols for identification and identifying humans as the species. Finally, we visualized the enrichment results using the R package (ggplot2).

### Signal pathway analysis

We obtained data from the UALCAN database to look into the participation of TRAP1 in the mammalian target of rapamycin (mTOR) and receptor tyrosine kinase (RTK) signaling pathways in normal and tumor tissues.

### Cell culture and stable cell line construction

Although the bioinformatic analysis in this study was based on human genomic data, the mouse hepatocellular carcinoma cell line Hepa1-6 was selected for in vitro functional validation, considering the highly conserved amino acid sequence of the TRAP1 protein across mammals. The liver cancer cell line Hepa1-6 was supplied by Wuhan Pricella Biotechnology Co., Ltd. (Wuhan, China), whereas the cryopreserved 293T cells in our laboratory were obtained from the Cell Resource Center (IBMS, CAMS/PUMC) of Peking Union Medical College. At 37 °C in a humidified incubator with 5% CO_2_, both cell lines were cultivated in complete Dulbecco's Modified Eagle Medium containing 0.1% β-mercaptoethanol, 1% non-essential amino acids, 10% fetal bovine serum (Gibco, USA), and 1% penicillin–streptomycin. Cells were regularly monitored and passaged at 80% confluence for optimal growth. To generate stable TRAP1 knockdown cell lines, we introduced TRAP1 shRNA (shTRAP1) into Hepa1-6 cells via lentiviral vectors, using a control vector as the negative control. Lentiviral packaging was done in 293T cells, and Hepa1-6 cells were infected with shTRAP1 or control vectors for 48 h in 6-well plates, followed by selection with 2 μg/mL puromycin for an additional 48 h. Western blot and reverse transcription quantitative polymerase chain reaction (RT-qPCR) were used to confirm successful transduction. The sequence of the shTRAP1 used was: 5′-GCTGACAAGGTTGAAGTCTATTTCAAGAGAATAGACTTCAACCTTGTCAGC-3′.

### RNA extraction and RT-qPCR

As directed by the manufacturer, total RNA was isolated using an RNA purification kit (Thermo Scientific, USA). A NanoDrop One/One C spectrophotometer (Thermo Scientific) was used to measure the RNA's concentration and purity. A kit called Takara's Reverse Transcription was used to synthesize cDNA. RT qPCR was conducted on a QuantStudio 3 system (Applied Biosystems, USA) to quantify TRAP1 and GAPDH expression levels, using GAPDH as the internal control. The primers used were as follows: GAPDH forward, 5'-AGCCACATCGCTCAGACAC-3' and reverse, 5'-TTAAAAGCAGCCCTGGTGAC-3'; TRAP1 forward, 5'-CAGGACAGTTATACAGCACACAG-3' and reverse, 5'-CTCATGTTTGGAGACAGAACCC-3'. The PCR settings were 50 °C for 2 min, 95 °C for 2 min, and 40 cycles of 95 °C for 15 s and 60 °C for 60 s. The 2^-ΔΔCt technique was used to measure the levels of gene expression.

### Protein separation and western blotting

Total protein was extracted from hepatocellular carcinoma (HCC) cell lines using radioimmunoprecipitation assay lysis buffer with 1% protease inhibitors. The Pierce BCA Protein Assay Kit (Thermo Scientific) was used to measure the protein concentration. Following their separation on a 10% sodium dodecyl sulfate–polyacrylamide gel electrophoresis gel, the proteins were moved onto a polyvinylidene fluoride membrane, blocked for 2 h using 5% nonfat milk, and then incubated for the entire night with antibodies against rabbit anti-TRAP1 (1:1000; Absin, China) and mouse anti-GAPDH (1:2000; Proteintech, China). The next day, membranes were incubated for 2 h with matching secondary antibodies (1:10,000; Thermo Scientific). For efficiency, the Western blot membranes were cut horizontally prior to antibody hybridization to allow simultaneous probing of the target protein and the loading control based on their respective molecular weights.

### Flow cytometric analysis of apoptosis

To accurately evaluate the effect of TRAP1 knockdown on apoptosis, flow cytometric analysis was performed using an Annexin V-fluorescein isothiocyanate (APC)/7-aminoactinomycin D (7-AAD) Apoptosis Detection Kit (BIOLEGEND Biotechnology Co., Ltd., California, USA). Briefly, shTRAP1 and control cells were trypsinized, collected, and washed twice with ice-cold PBS. Subsequently, 1 × 10^6^ cells per sample were resuspended in 100 μL of 1 × Binding Buffer, followed by the addition of 5 μL Annexin V-APC and 5 μL 7-AAD staining solutions. After gentle mixing, the cells were incubated at room temperature in the dark for 15 min. Finally, 400 μL of 1 × Binding Buffer was added, and the samples were immediately analyzed using a flow cytometer (BD FACSCelesta™, BD Biosciences, USA). The experiment was independently repeated three times. Cells positive for Annexin V-APC and negative for 7-AAD were considered early apoptotic, while cells positive for both Annexin V-APC and 7-AAD were identified as late apoptotic or necrotic.

### ROS detection

The 2′,7′-dichlorodihydrofluorescein diacetate (H2-DCFDA) assay and a ROS detection kit (Beyotime Biotechnology Co., Ltd., Shanghai, China) were used to measure ROS production in liver hepatocellular carcinoma (LIHC) cells. After resuspending LIHC cells in a culture medium at a density of 5 × 10^4^ cells/mL, 100 µL was added to each well of a 96-well plate, and the cells were incubated for 8 h. The cells were then rinsed three times with a serum-free medium. A microplate reader with excitation at 488 nm and emission at 525 nm was used to measure the ROS levels after cells were treated with 10 µM H2-DCFDA for 25 min at 37 °C in the dark.

### Cell counting kit-8 (CCK-8) assay

While shTRAP1 and control cells were being cultivated under optimal conditions, the material was centrifuged for 3 min at 1000 rpm. After discarding the supernatant, the cell pellet was resuspended in 1 mL of growth medium. In a counting chamber, 900 µL of 1 × phosphate buffered saline (PBS) was combined with a 100 µL aliquot of this suspension, and a Vi-Cell BLU Cell Viability Analyzer (Beckman Coulter, Inc., Brea, CA, USA) was used to count the cells. The corrected cell density was 4 × 10^4^ cells/mL. The cell pellet was resuspended in 1 mL growth media after the supernatant was disposed of, and 10 µL of CCK8 solution was added to each well. To assess the effect of TRAP1 knockdown on cell growth, wells were incubated for 4 h at 37 °C in the dark. Absorbance at 450 nm was quantified using a microplate reader.

### Wound healing assay

In a 6-well plate, 5.0 × 10^5^ shTRAP1 and control cells were seeded and grown to 80% confluence. A 200 µL pipette tip created a wound in each well for migration and invasion studies. After three PBS washes to remove detached cells, a serum-free medium was added. Microscope images (LEICA, Germany) were taken at 0 and 24 h and processed with ImageJ to assess TRAP1 knockdown effects on cell migration and invasion.

### Transwell cell invasion assay

To evaluate the effect of TRAP1 knockdown on the invasive ability of hepatocellular carcinoma cells, a Transwell invasion assay was performed. Matrigel matrix (Corning, USA) was diluted with serum-free cold DMEM medium at an 8:1 ratio, and 50 μL of the mixture was evenly coated onto the upper polycarbonate membrane of a Transwell insert (8 μm pore size, Corning), followed by incubation at 37 °C for 1 h to solidify. Cells from the shTRAP1 and control groups were resuspended in serum-free medium at a density of 1 × 10^5^ cells/mL. A total of 500 μL of cell suspension was added to the upper chamber, while the lower chamber was filled with 800 μL of complete medium containing 10% FBS as a chemoattractant. Following 24 h of incubation at 37 °C with 5% CO₂, non-invaded cells and Matrigel on the upper surface were gently removed using a cotton swab. Invaded cells on the lower membrane were fixed with 4% paraformaldehyde for 30 min and stained with 0.1% crystal violet for 15 min. Five randomly selected fields per membrane were imaged and counted under an inverted microscope (LEICA, Germany) to quantitatively analyze cell invasive capability.

### Statistical analysis

Every cellular experiment was performed in three copies. GraphPad Prism 10.0 and R version 2024.04.2 (https://www.r-project.org/) were used to analyze the data. Data is presented using the means ± standard error of the means. The continuous data between the two groups were compared using the student's t-test. Unpaired Wilcoxon rank-sum tests and signed rank tests were used to analyze the expression differences between normal and malignant samples within each tumor. The assumptions for the survival analysis were tested using the log-rank test. The relationship between gene expression and immune infiltration ratings for each tumor was assessed using Pearson's correlation coefficients. A significance level of *p* < 0.05 was established.

## Results

### TRAP1 expression and subcellular localization in normal human tissues

**Fig. 1:**
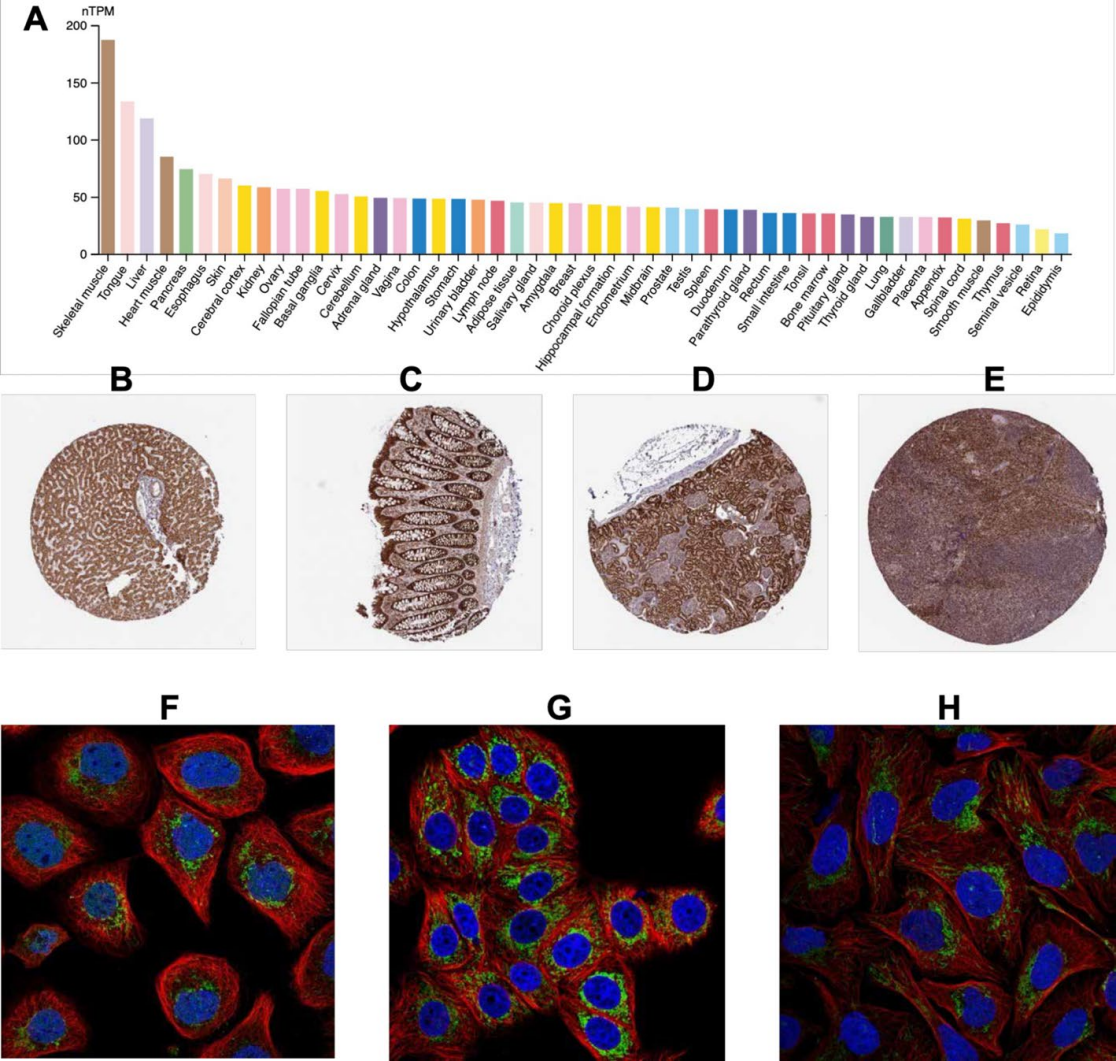
TRAP1 expression and subcellular localization across different normal human tissues. **a** TRAP1 expression levels in normal tissues based on data from the HPA (N = 13,084). **b**–**e** Representative immunohistochemistry (IHC) images showing TRAP1 expression in normal tissues of the liver, colon, kidney, and tonsils. **f**–**h** Subcellular localization of TRAP1 as observed in the HPA: **f** A-431 cell line, **g** MCF-7 cell line, and **h** U2OS cell line

Skeletal muscle, tongue, liver, heart muscle, and pancreas all showed significantly greater levels of TRAP1 expression (Fig. 1a). Immunohistochemistry showed representative staining in the liver, colon, kidney, and tonsils (Fig. 1b–e), whereas immunofluorescence indicated predominant TRAP1 localization in the mitochondria and nucleoplasm (Fig. 1f–h).

### TRAP1 expression in various tumor tissues

**Fig. 2:**
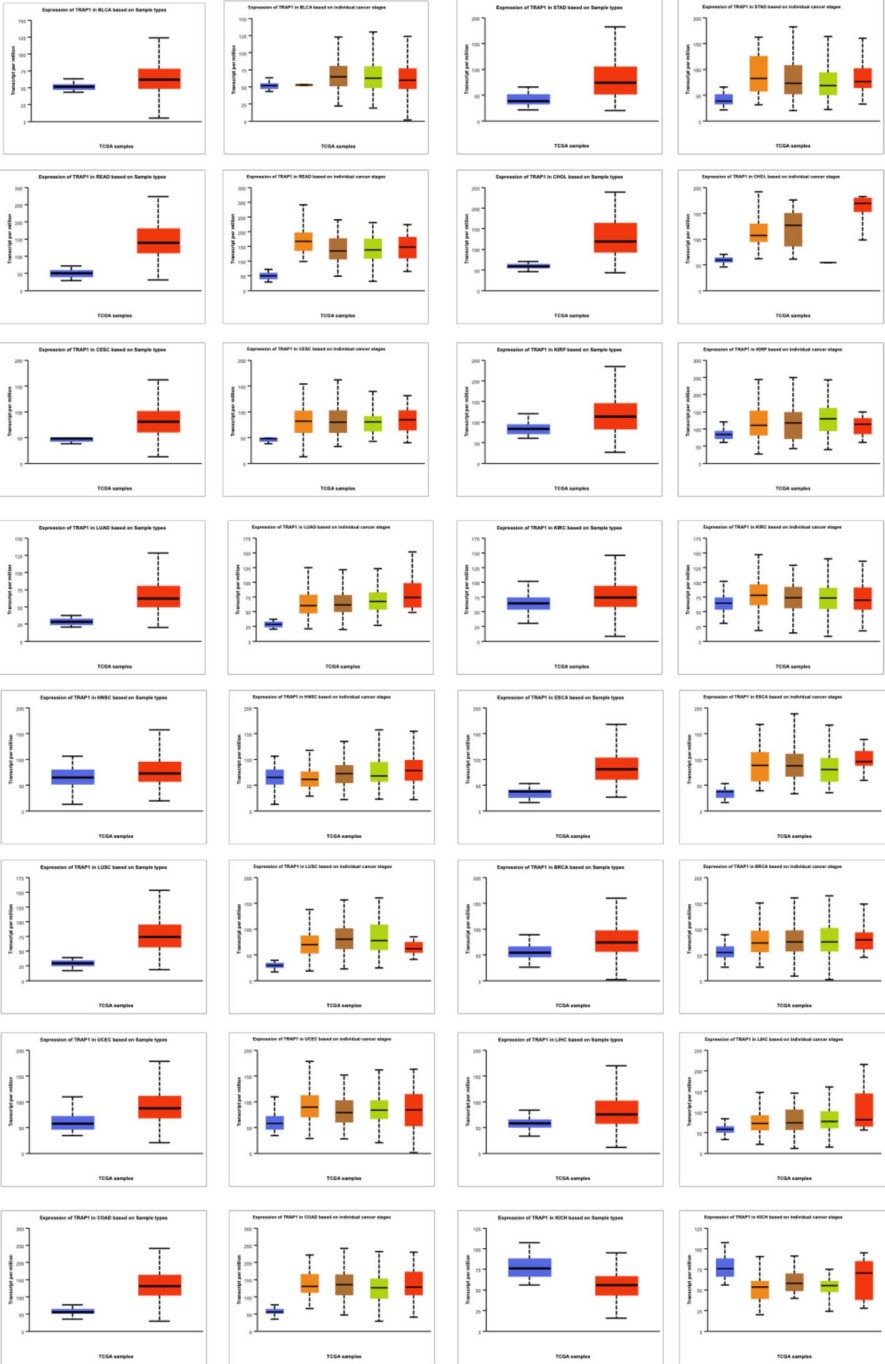
The TCGA dataset demonstrated TRAP1 mRNA expression levels in normal (blue) and malignant (red) tissues, as well as its expression across distinct clinicopathological stages

Significantly upregulated TRAP1 mRNA expression was observed in 15 types of tumor tissues, including bladder urothelial carcinoma (BLCA), STAD, lung adenocarcinoma (LUAD), head and neck squamous cell carcinoma (HNSC), and LIHC (Online Resource 2), while its expression was notably downregulated in kidney chromophobe carcinoma (KICH). Based on the UALCAN database analysis, TRAP1 expression levels significantly differed across various clinical stages of STAD, LUAD, kidney renal clear cell carcinoma (KIRC), HNSC, and LIHC (Fig. 2).

### TRAP1 expression is significantly associated with prognosis

**Fig. 3:**
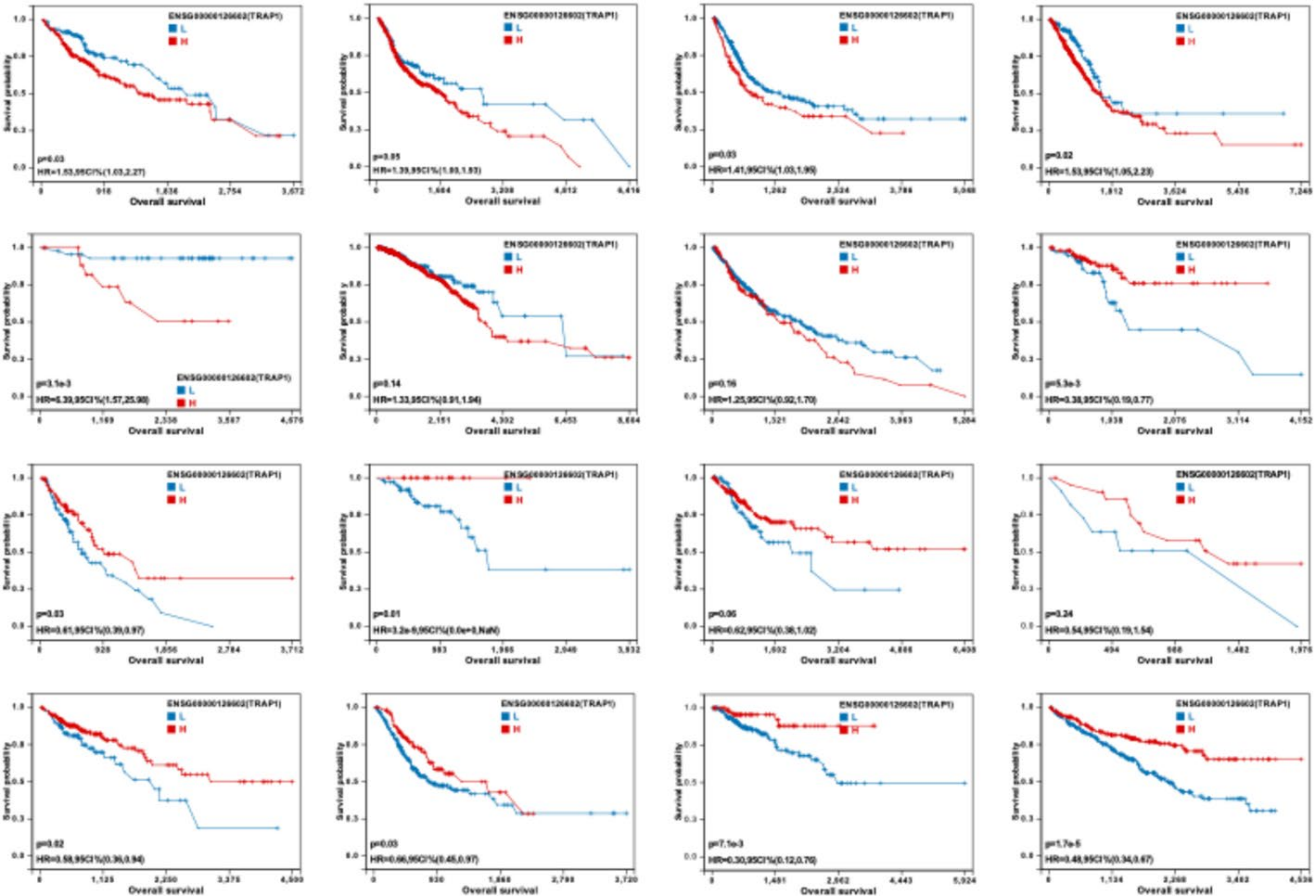
Kaplan–Meier survival analysis curves for the TRAP1 high- and low-expression groups

Analysis of TCGA data revealed that high TRAP1 expression was significantly associated with shortened median overall survival (OS) in patients with LIHC, BLCA, LUAD, and KICH, while it correlated with prolonged median OS in patients with uterine corpus endometrial carcinoma (UCEC), ESCA, READ, COAD, STAD, kidney renal papillary cell carcinoma (KIRP), and KIRC (Fig. 3).

### TRAP1 gene exhibits variations across multiple cancers

**Fig. 4:**
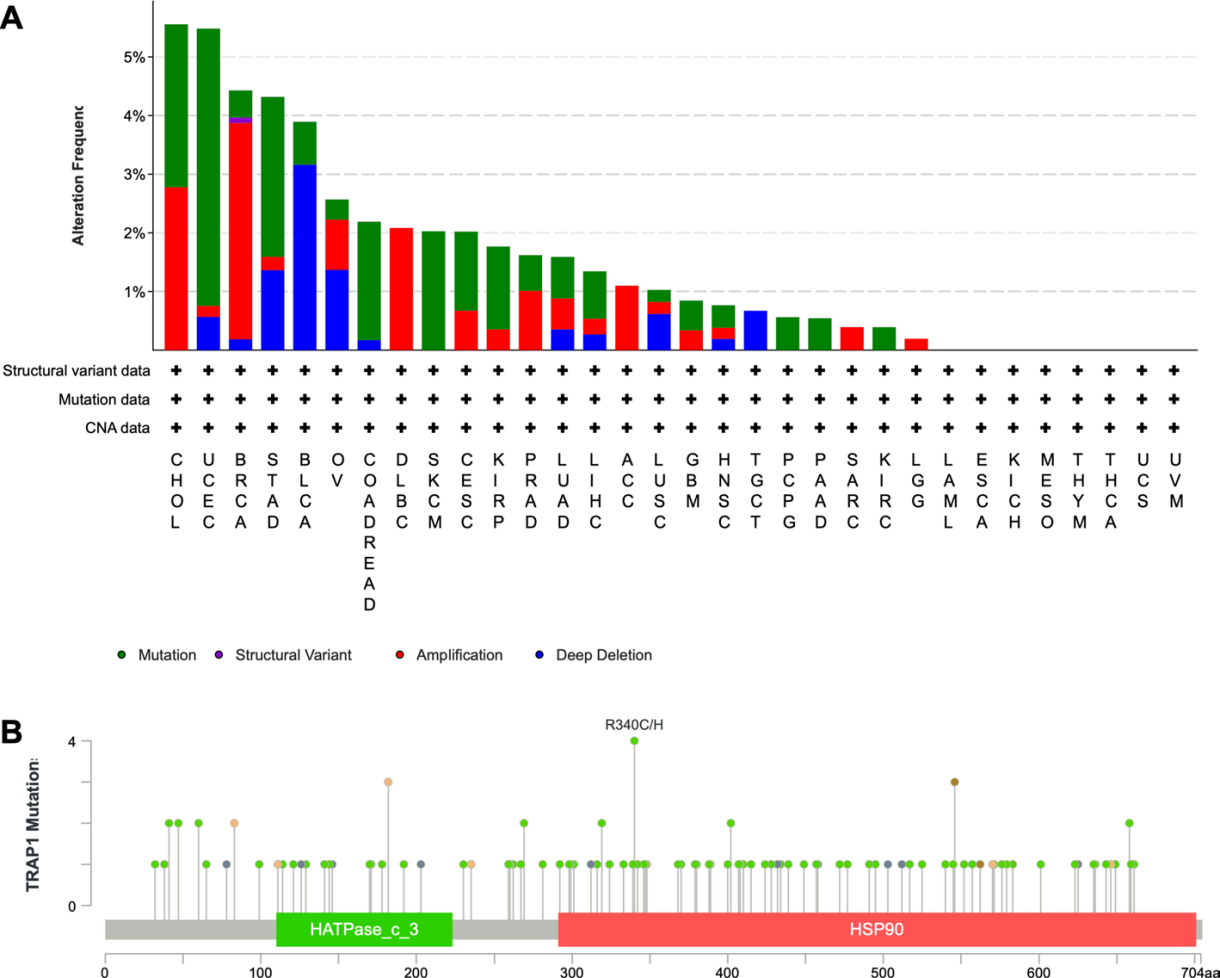
Mutation characteristics of TRAP1 in different tumors. **a** Analysis of TRAP1 alterations, with green representing mutations, purple for structural variations, red for amplifications, and blue for deep deletions. **b** Mutation sites and types of TRAP1

Data from the cBioPortal revealed significant alterations in the TRAP1 gene across various cancers, with "mutations" being the most notable. Specifically, cholangiocarcinoma showed the highest frequency of TRAP1 alterations, followed by UCEC. Notably, in skin cutaneous melanoma (SKCM), pheochromocytoma and paraganglioma (PCPG), and PRAD, TRAP1 alterations were predominantly of the "mutation" type. In diffuse large B-cell lymphoma (DLBC), adrenocortical carcinoma (ACC), sarcoma (SARC), and lower grade glioma (LGG), the alterations were of the “amplification” type, whereas in testicular germ cell tumors, they were characterized by “deep deletions” (Fig. 4a). A detailed analysis of TRAP1 mutation sites and types revealed that 86 of the 112 mutation samples were missense mutations. The R340C/H site showed the highest mutation frequency in UCEC, STAD, and COADREAD (Fig. 4b). Online Resource 3 offers comprehensive details on the different types of mutations.

### Differences in TRAP1 DNA methylation between tumor and normal tissues

One important epigenetic process that modifies the transcription of genes is DNA methylation [16]. Across a range of cancer types, tumor tissues had much greater levels of TRAP1 DNA methylation than normal tissues, including breast invasive carcinoma (BRCA), HNSC, KIRC, KIRP, lung squamous cell carcinoma (LUSC), and PRAD, using the UALCAN database. In contrast, TRAP1 methylation levels were significantly decreased in the THCA group (Online Resource 4).

### TRAP1 expression levels in relation to immune checkpoint genes and immune cell infiltration

**Fig. 5:**
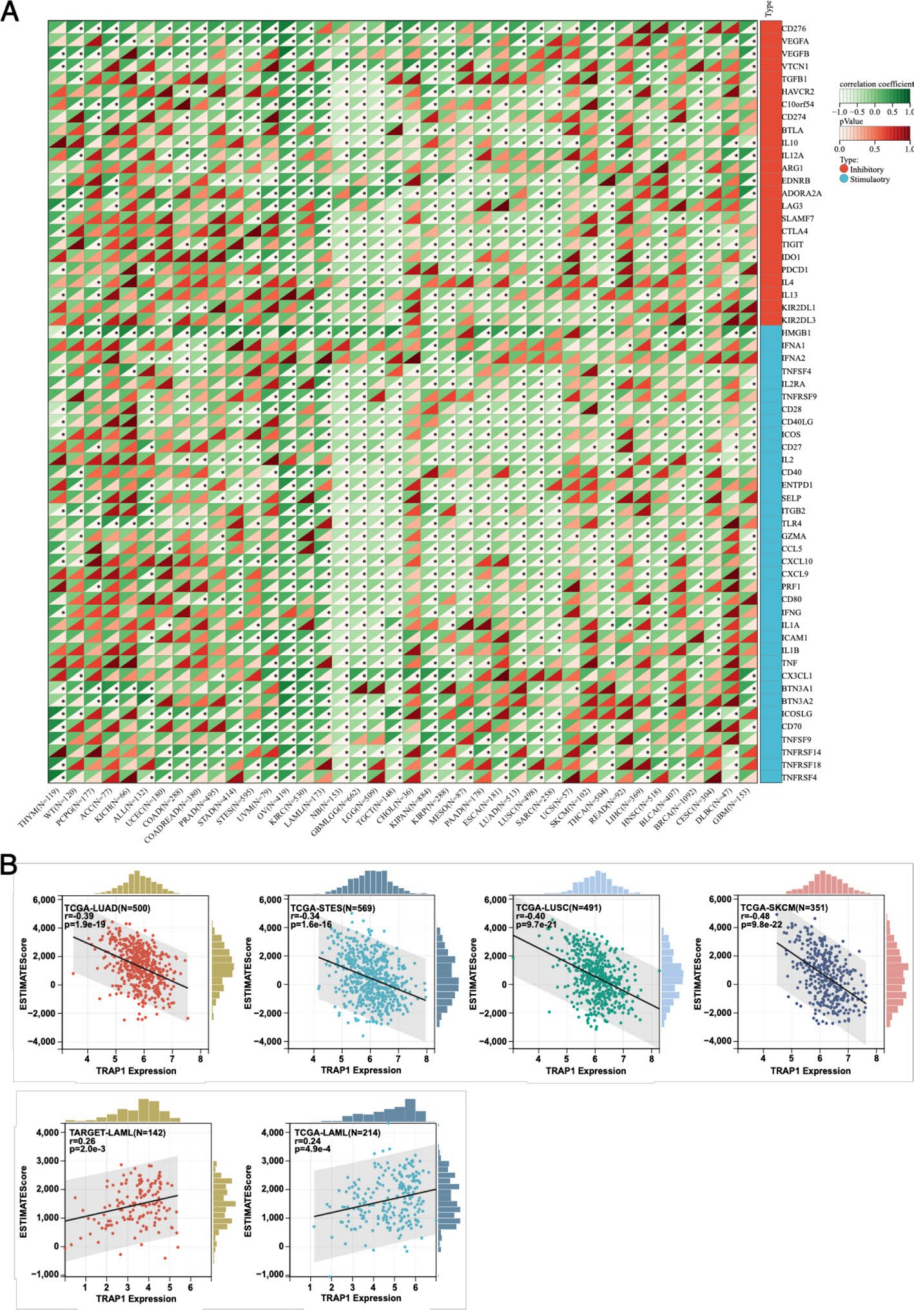
TRAP1 expression and tumor immune infiltration. **a** Correlation analysis between TRAP1 expression levels and immune checkpoint genes. **b** Scatter plot of TRAP1 expression versus tumor immune infiltration

TRAP1 expression correlated positively with most ICP genes in WT, PCPG, KICH, ACC, and UCEC but negatively in NB and GBMLGG (Fig. 5a). Further investigations indicated TRAP1's connection to immune infiltration in 34 cancer types (Online Resource 5). Notably, 32 cancers, including TCGA-LUAD, TCGA-STES, TCGA-LUSC, and TCGA-SKCM, exhibited significant negative correlations, whereas TARGET-LAML and TCGA-LAML showed positive correlations (Fig. 5b).

### Enrichment analysis of TRAP1-related genes and proteins

**Fig. 6:**
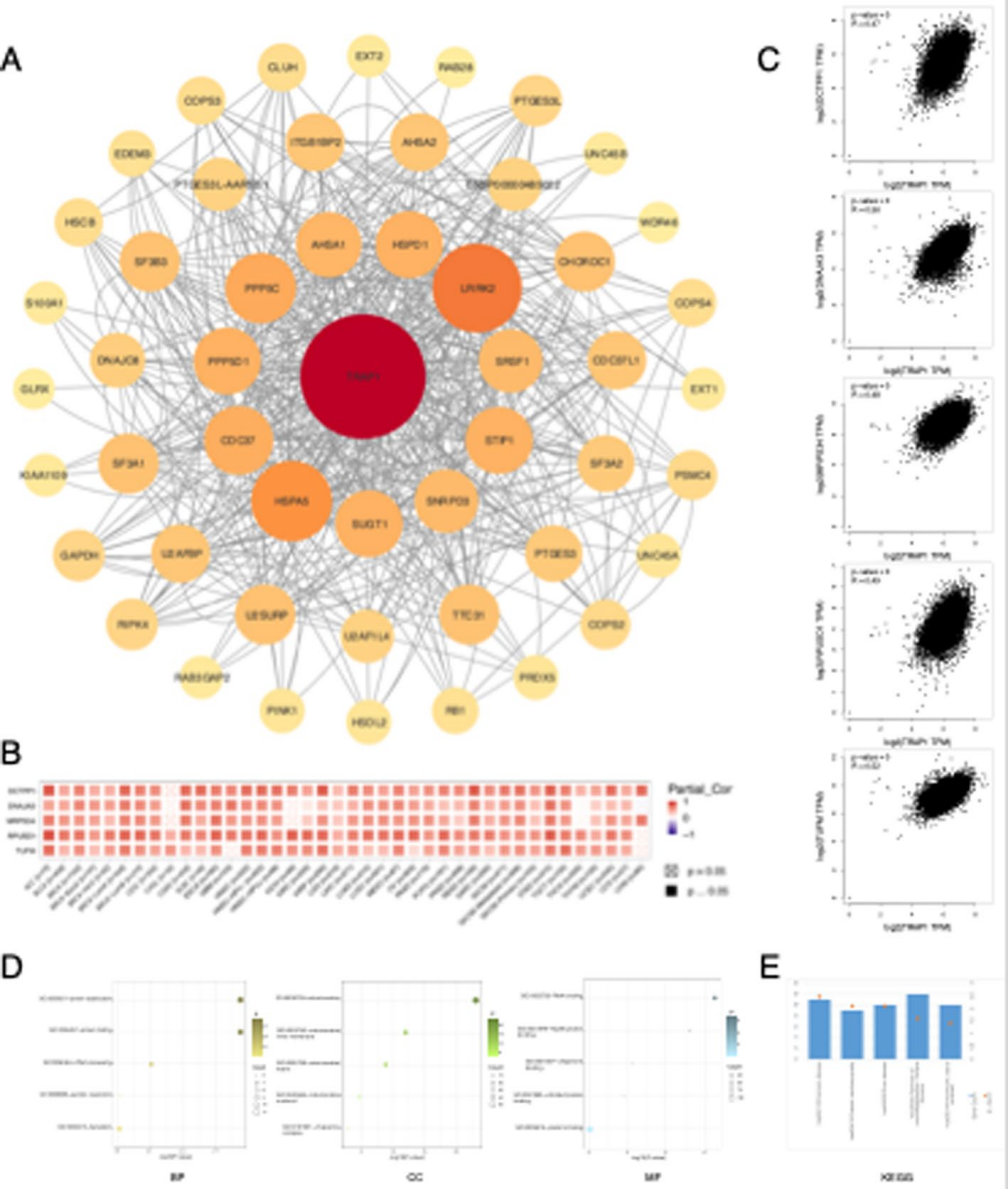
Enrichment analysis of TRAP1-related genes and proteins. **a** The STRING database was used to create the TRAP1 protein–protein interaction network. **b** Heatmap showing the expression correlation between TRAP1 and selected target genes in pan-cancer. **c** Scatter plot illustrating the correlation between TRAP1 and selected target genes. **d** analysis of GO enrichment. MF: Molecular Function; CC: Cellular Component; BP: Biological Process. (Larger dots represent more genes, whereas darker colors represent more significant *p*-values). **e** KEGG enrichment histogram (the vertical axis represents the gene counts, and the dot size corresponds to p-value significance)

Figure 6a lists the top 50 proteins interacting with TRAP1. Online Resource 6 reports the top 100 genes interacting with TRAP1. As some genes were not included in the TIMER 2.0 database, we selected 5 out of the top 7 interacting genes for analysis. In the TCGA dataset, RPUSD1, MRPS34, DNAJA3, TUFM, and DCTPP1 showed significant positive correlations with TRAP1 (Fig. 6b, c). Gene ontology enrichment analysis indicated multiple TRAP1-related biological processes, including protein stabilization and folding, ribosomal RNA processing, and aerobic respiration. The associated cellular component terms involved mitochondrial structures, such as matrix and nucleoid. Molecular function terms encompassed RNA and Hsp90 protein binding. Figure 6d details gene counts for the top 18 biological terms selected by ascending p-values; Fig. 6e shows similar data for the top six KEGG pathways. TRAP1 was implicated in critical processes and pathways like Parkinson's disease and diabetic cardiomyopathy.

### Signal pathway analysis

**Fig. 7:**
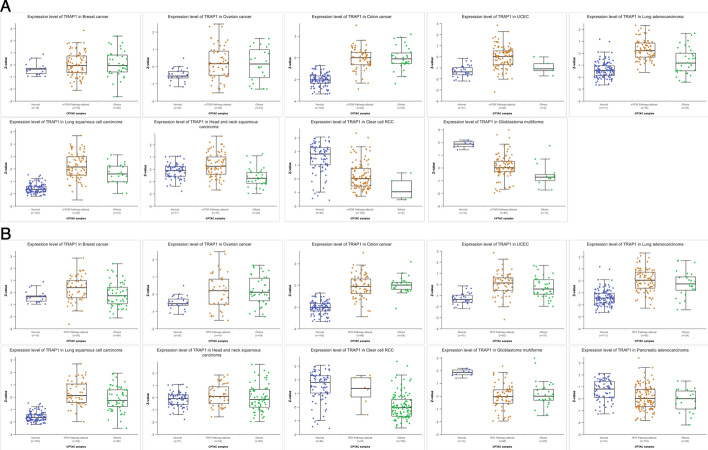
Signal pathway analysis. **a** mTORC signaling pathway analysis. Blue represents normal; orange indicates mTOR pathway-altered; green represents other alterations. **b** RTK signaling pathway analysis. Blue represents normal; orange represents mTOR pathway-altered; green represents other alterations

According to data from the UALCAN database, malignancies such as endometrial, breast, ovarian, colon, HNSC, LAUD, and LUSC increased TRAP1 expression, which is associated with the control of the mTOR pathway (Fig. 7a). In contrast, clear cell RCC and GBM exhibit reduced TRAP1 involvement in the mTOR pathway. Similarly, these cancers—excluding pancreatic cancer—also demonstrate high TRAP1 expression, impacting the RTK-RAS-MAPK pathway. In GBM and PRAD, however, TRAP1 shows decreased pathway involvement. Clear cell RCC showed no significant differences, possibly owing to small sample sizes (Fig. 7b).

### Functional validation of TRAP1 in liver cancer cell lines

**Fig. 8:**
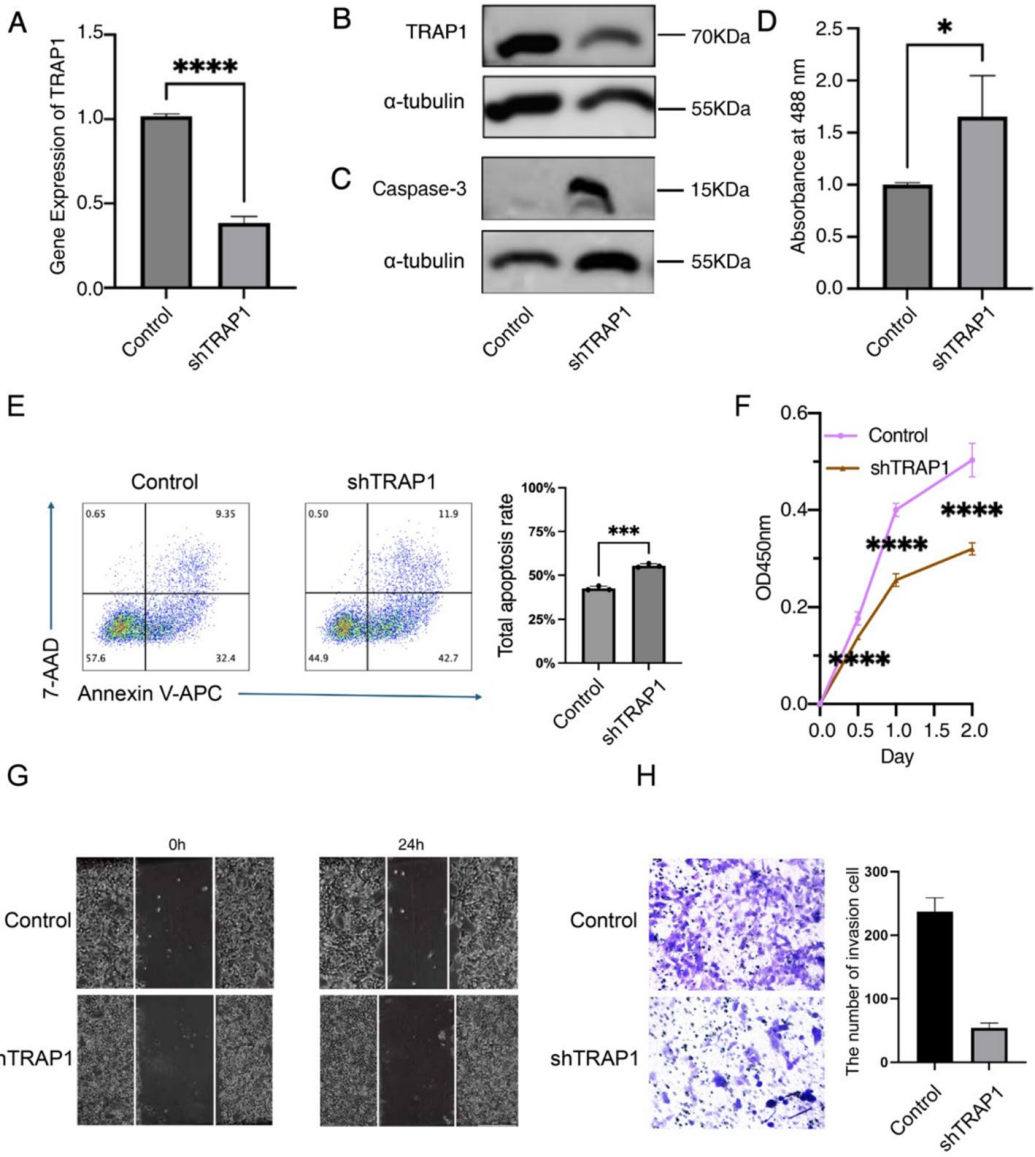
Effects of TRAP1 on apoptosis, proliferation, and migration in liver cancer. **a** RT-qPCR validation of TRAP1 knockdown efficiency. **b** Western blot validation of TRAP1 knockdown efficiency. **c** Effect of TRAP1 knockdown on apoptosis-related proteins in Hepa1-6 cells. **d** Impact of TRAP1 knockdown on reactive oxygen species (ROS) generation in Hepa1-6 cells. **e** Flow cytometry analysis of the effect of TRAP1 knockdown on apoptosis in Hepa1-6 cells. **f** The CCK-8 experiment demonstrates the effects of TRAP1 knockdown on the 24-h proliferation of Hepa1-6 cells. **g** Assay for wound healing assessing how TRAP1 knockdown affects the migration of Hepa1-6 cells. **h** Transwell assay to detect the effect of TRAP1 knockdown on the invasion of Hepa1-6 cells

We validated TRAP1's role in HCC using Hepa1-6 cells, achieving a 62.8% knockdown efficiency via small hairpin RNA (shRNA) lentivirus, confirmed by western blot analysis (Fig. 8a, b). Results from the ROS assay indicated that intracellular ROS levels were significantly elevated following TRAP1 knockdown (Fig. 8c), suggesting that loss of TRAP1 induced substantial oxidative stress. To investigate whether this oxidative stress ultimately led to apoptosis, we examined the activity of the key apoptosis executor caspase-3 and observed a marked increase in its level (Fig. 8d). To assess the proportion of apoptotic cells further quantitatively, Annexin V-APC/7-AAD double-staining flow cytometry was performed. The results revealed that the total apoptosis rate in the shTRAP1 group was significantly increased by 13.02% compared with the control group (Fig. 8e), convincingly demonstrating that TRAP1 knockdown induces apoptosis. TRAP1 knockdown reduced HCC cell proliferation according to CCK-8 assays (Fig. 8f), and significantly hindered cell migration in wound-healing assays (Fig. 8g). Transwell invasion assays revealed that the number of cells invading through the Matrigel-coated membrane was significantly reduced in the shTRAP1 group compared with the control group, indicating that TRAP1 knockdown effectively suppressed the invasive ability of hepatocellular carcinoma cells (Fig. 8h). These results demonstrate that TRAP1 knockdown triggers ROS-mediated oxidative stress, subsequently activating caspase-3 and ultimately leading to apoptosis, while concurrently inhibiting the proliferative, migratory, and invasive capacities of HCC, affirming its potential as an oncogene.

## Discussion

With increasing incidence and mortality rates, cancer has emerged as a major cause of death worldwide, posing a serious threat to public health [4, 17]. Worldwide, cancer cases are projected to increase by 47% from 2020 levels, reaching 28.4 million by 2040 [18]. Despite advances in immunotherapy and biomarker research for improving cancer diagnosis, treatment, and prognosis, identifying novel prognostic biomarkers remains essential for cancer prevention and therapy. The pan-cancer analysis is an effective approach for discovering such biomarkers and therapeutic targets. In this study, we considered TRAP1 a potential broad-spectrum therapeutic target through extensive database analysis. To validate this hypothesis at the functional level, we selected hepatocellular carcinoma (HCC)—which demonstrated significantly elevated TRAP1 expression and was associated with poor prognosis in the pan-cancer analysis—as a model for subsequent mechanistic exploration.

TRAP1 encodes a mitochondrial chaperone protein within the HSP90 family. In healthy cells, TRAP1 enhances oxidative metabolism and facilitates programmed cell death [19], with high expression in tissues like skeletal muscle, tongue, liver, heart muscle, and pancreas. However, dysregulated TRAP1 expression is common in cancers, where it promotes tumor growth by enhancing glycolytic metabolism, inhibiting apoptosis, and regulating mitochondrial permeability [20].

Owing to the metabolic properties of tumors, TRAP1 plays a dual role in human cancers, acting as a tumor suppressor and an oncogene [15], as supported by our analysis of the SangerBox database. Immunohistochemistry has shown significantly higher TRAP1 expression in colorectal cancer [9], ovarian cancer [21], and LUAD tissues [22], consistent with our results. The UALCAN database further revealed variations in TRAP1 expression across different pathological stages of STAD, LUAD, KIRC, HNSC, and LIHC. Additionally, studies have demonstrated a strong correlation between TRAP1 expression, lymph node metastases, and lung cancer stage [22]. These results have been confirmed in HNSC and gastric cancer [23]. These findings underscore TRAP1's critical function in carcinogenesis and disease progression, indicating significant expression differences in malignant versus normal tissues in numerous cancers.

Survival analysis based on TCGA data demonstrated that patients with high TRAP1 expression in LIHC, BLCA, LUAD, and KICH tissues exhibited significantly shortened median survival, while median survival increased with elevated TRAP1 expression in UCEC, ESCA, READ, COAD, STAD, KIRP, and KIRC tissues. These findings indicate that aberrant TRAP1 expression can be a significant prognostic marker for specific cancer types. However, the database analysis did not adjust for confounding factors such as sex, age, and tumor grade, necessitating further clinical studies to validate these findings.

Genetic mutations in DNA have been demonstrated to be important for the development and spread of malignancies [24, 25]. Utilizing the cBioPortal database, we evaluated genetic variations in TRAP1 across various cancers. TRAP1 exhibits genetic variations in most cancers, the “mutation” type variation is particularly common. The highest frequency of TRAP1 mutations (4.73%) was observed in patients with UCEC. TRAP1 amplification was most frequent in breast-invasive carcinoma (3.69%). In BLCA, the prevalence of TRAP1 deep deletions was 3.16%. Conversely, the “structural variation” type was relatively rare. We also identified R340C/H as the most common mutation site, with four samples (two from endometrial cancer, one from gastric cancer, and one from colorectal cancer) exhibiting mutations at this site.

DNA methylation participates in cancer development [26]. Our analysis revealed elevated TRAP1 methylation levels in most cancers, with a notable decrease in thyroid cancer. Reduced methylation can lead to genomic instability, increasing tumor heterogeneity and promoting cancer progression [3]. This may explain the decreased TRAP1 methylation observed in thyroid cancer. According to our research, TRAP1 methylation might be a useful target for therapy.

The tumor microenvironment (TME) has been gaining attention as a critical element in cancer research and treatment. Interactions between tumor cells and their surrounding environment within the TME significantly affect cancer development, metastasis, and response to treatment [27]. ICPs and immune-infiltrating cells are key factors determining tumor progression and treatment response within the TME [28, 29], and inhibitors targeting ICPs have shown significant therapeutic efficacy [30, 31]. Using the SangerBox database to investigate TRAP1’s correlation with common ICPs, we found positive TRAP1 expression correlation with most ICP genes in malignancies like WT, PCPG, KICH, ACC, and UCEC while showing inverse correlations in NB and GBMLGG. This suggests that TRAP1 may influence tumor development and progression by regulating ICP genes and thus impact treatment responses and prognoses, highlighting its dual role in tumors [15]. Additionally, we analyzed TRAP1’s link with immune cell infiltration across 34 cancer types and found significant negative correlations in 32 types and positive correlations in two, indicating TRAP1’s potential role in modulating immune infiltration. TRAP1 in serum also demonstrated strong diagnostic value for small cell lung cancer, likely linked to immune cell secretion into circulation [32]. These results underscore TRAP1's association with immune infiltration and potential as a cancer immunotherapy target.

Further enrichment analysis revealed that TRAP1 was significantly and positively correlated with several proteins, including DNAJA3, TUFM, RPUSD1, MRPS34, and DCTPP1, and all are closely related to cellular metabolism and mitochondrial function. TRAP1 controls the production of ROS and mitochondrial OXPHOS, reducing oxidative stress and shielding cancer cells from harm caused by ROS [33, 34]. Lower ROS levels are linked to increased TRAP1 expression, which encourages metabolic reprogramming and apoptosis evasion in cancer cells [6]. TRAP1 probably has beneficial physiological effects by controlling mitochondrial respiration, maintaining mitochondrial homeostasis, and balancing OXPHOS and glycolysis [35]. Furthermore, TRAP1 is involved in a number of signaling networks, including the RTK-RAS-MAPK and mTOR pathways. mTOR signaling controls many important physiological processes, including protein synthesis, cell division, metabolism, survival, catabolism, and autophagy. Age, metabolic disorders, and cancer have all been associated with impaired mTOR signaling [36–38], and mTOR activation promotes tumor growth and metastasis [39]. The RTK-RAS-MAPK signaling pathway is also critical for cell proliferation, and its dysregulation often leads to tumor development and progression [40, 41]. Moreover, studies have highlighted the significance of mitochondrial interactions between ERK1/2 and TRAP1 in cancer development, suggesting that TRAP1 may act as an oncogenic metabolic effector in the RAS/ERK signaling pathway [42]. Elevated TRAP1 expression is noted in the mTOR and RTK-RAS-MAPK pathways across various cancers. Oligodendrogliomas, however, exhibit reduced TRAP1 in both pathways, whereas clear cell RCC shows TRAP1 reduction primarily in the mTOR pathway, with minimal RTK-RAS-MAPK impact, likely due to sample limitations. We suggest TRAP1's pathway roles vary among cancers, influenced by immunological and metabolic profiles, requiring further experimentation. In tumor cell mitochondria, TRAP1 aids apoptosis evasion and regulates metabolic reprogramming, positioning it as a potential anticancer target due to its role in cellular metabolism and mitochondrial apoptosis control [20]. Disrupting TRAP1 activity may cause tumor cells to perish while having little effect on healthy cells [43]. TRAP1 inhibitors selectively affect cancer cells, making them a promising candidate strategy for cancer treatment [6].

In vitro, we found that TRAP1 deletion enhanced ROS generation, subsequently inducing apoptosis through activation of the caspase-3 pathway, while also reducing liver cancer cell invasion, migration, and proliferation. These findings highlight TRAP1’s role in cancer management, provide insights into liver cancer pathogenesis, and suggest opportunities for treatment improvements. Future research should investigate TRAP1’s impact on tumor growth. Despite a comprehensive analysis, limitations included our in vitro functional experiments utilized the mouse hepatocellular carcinoma cell line Hepa1-6, whereas the bioinformatic analysis was based on human cancer databases. Although TRAP1 is highly conserved across species and mouse models are widely employed in cancer research, such cross-species extrapolation requires further validation in future studies using additional human-derived liver cancer cell lines (e.g., Huh-7, HepG2) or patient-derived xenograft (PDX) models. Such efforts would strengthen the generalizability of our findings and provide direct support for TRAP1’s potential as a pan-cancer therapeutic target in humans. Thus, small clinical samples and data gaps in certain cancers. Additionally, some results depend on single methodologies or databases without cross-validation. Although we examined TRAP1 phenotypically, more research is required to validate its molecular mechanisms and biological functions.

In conclusion, the pan-cancer analysis in this study systematically delineated the clinical and molecular landscape of TRAP1 in human cancers, highlighting its broad involvement. Subsequent experimental validation in an HCC model demonstrated the critical role of TRAP1 in regulating cell proliferation, apoptosis, migration, and invasion. Together, these findings indicate that TRAP1 is a molecule with significant translational potential, particularly in liver cancer. Although the specific functions of TRAP1 in other cancer types warrant further investigation, this study lays a solid foundation for its future exploration as a pan-cancer target and provides compelling experimental evidence to support the development of TRAP1-targeted therapies for hepatocellular carcinoma.

## Supplementary Information

Supplementary Material 1.

Supplementary Material 2.

Supplementary Material 3.

Supplementary Material 4.

Supplementary Material 5.

Supplementary Material 6.

Supplementary Material 7.

Supplementary Material 8.

Supplementary Material 9.

Supplementary Material 10.

Supplementary Material 11.

## Data Availability

The datasets generated during and/or analyzed during the current study are available from the corresponding author on reasonable request.
