# Supplementary material for: Comprehensive pan-cancer analysis of TRAP1 and its experimental validation in hepatocellular carcinoma
Source: Discov Oncol. 2025 Dec 20;17:152. doi: 10.1007/s12672-025-04238-9 (PMC12835488; doi:10.1007/s12672-025-04238-9)
Supplement: Supplementary file 4 — Supplementary Material 4. [file 12672_2025_4238_MOESM4_ESM.docx]

A box plot showing the levels of TRAP1 DNA methylation in tumor and normal tissues, where red denotes tumor tissues and blue denotes normal tissues.


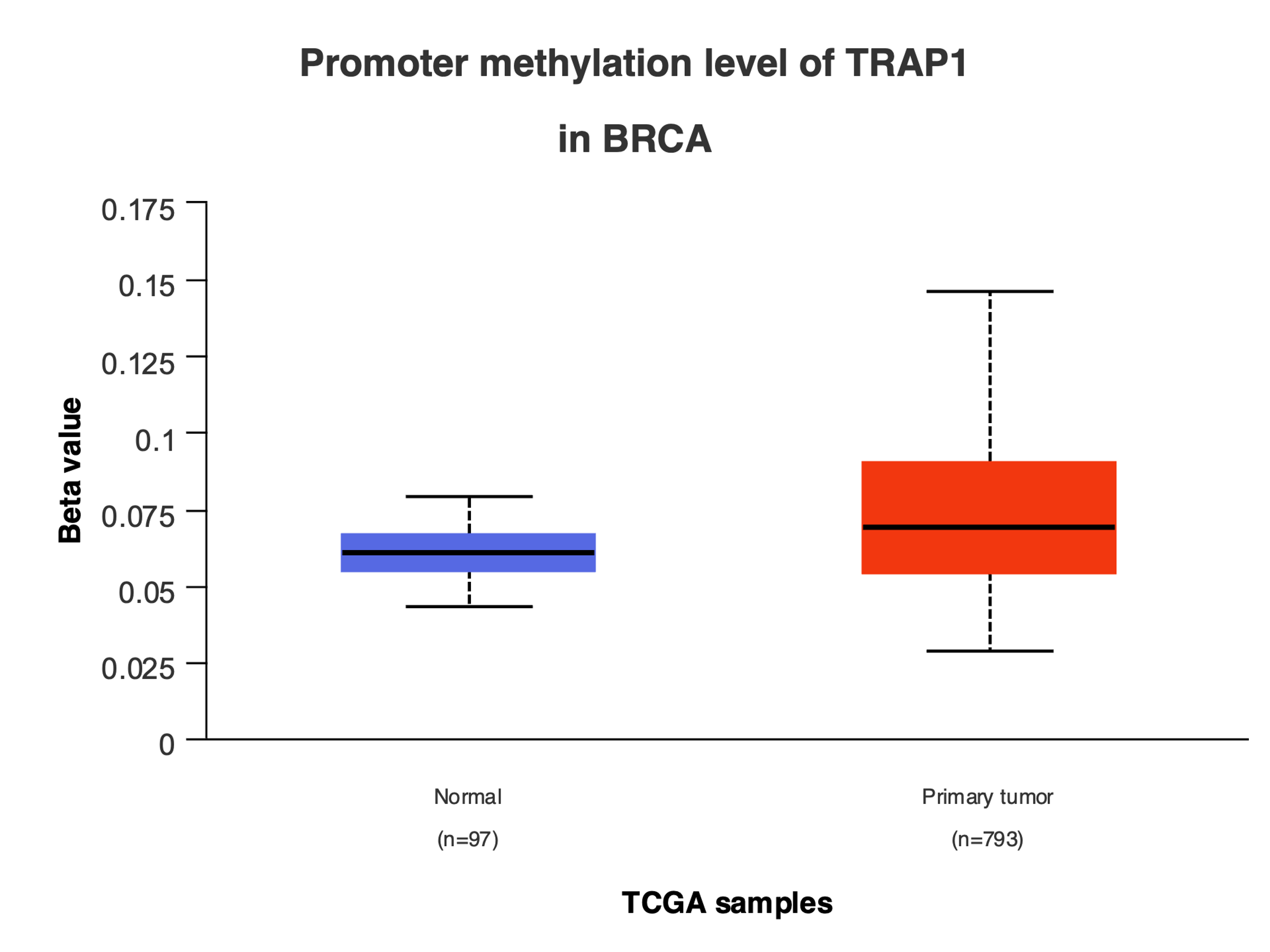


p=6.828e-08


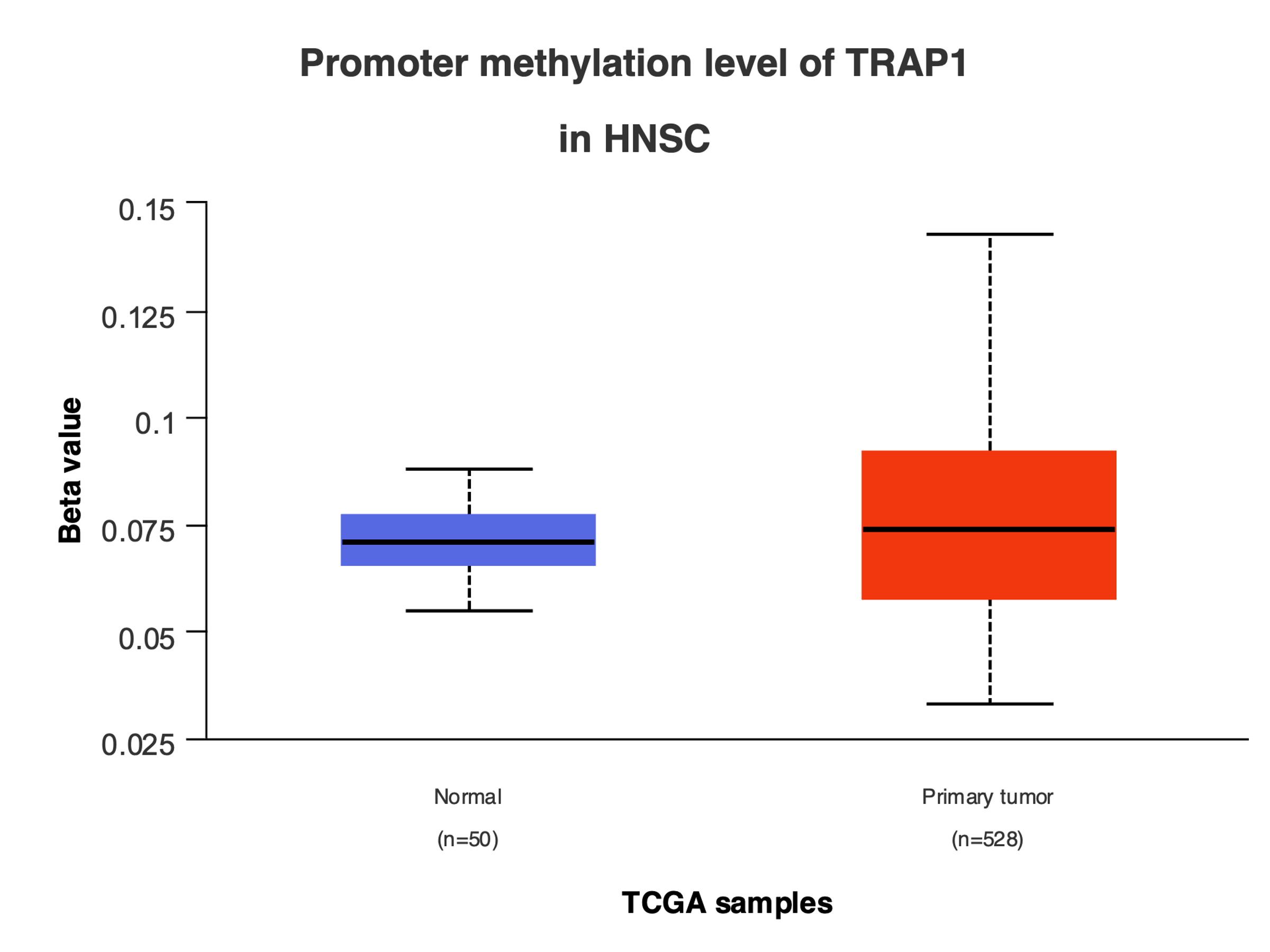


p=1.825e-04


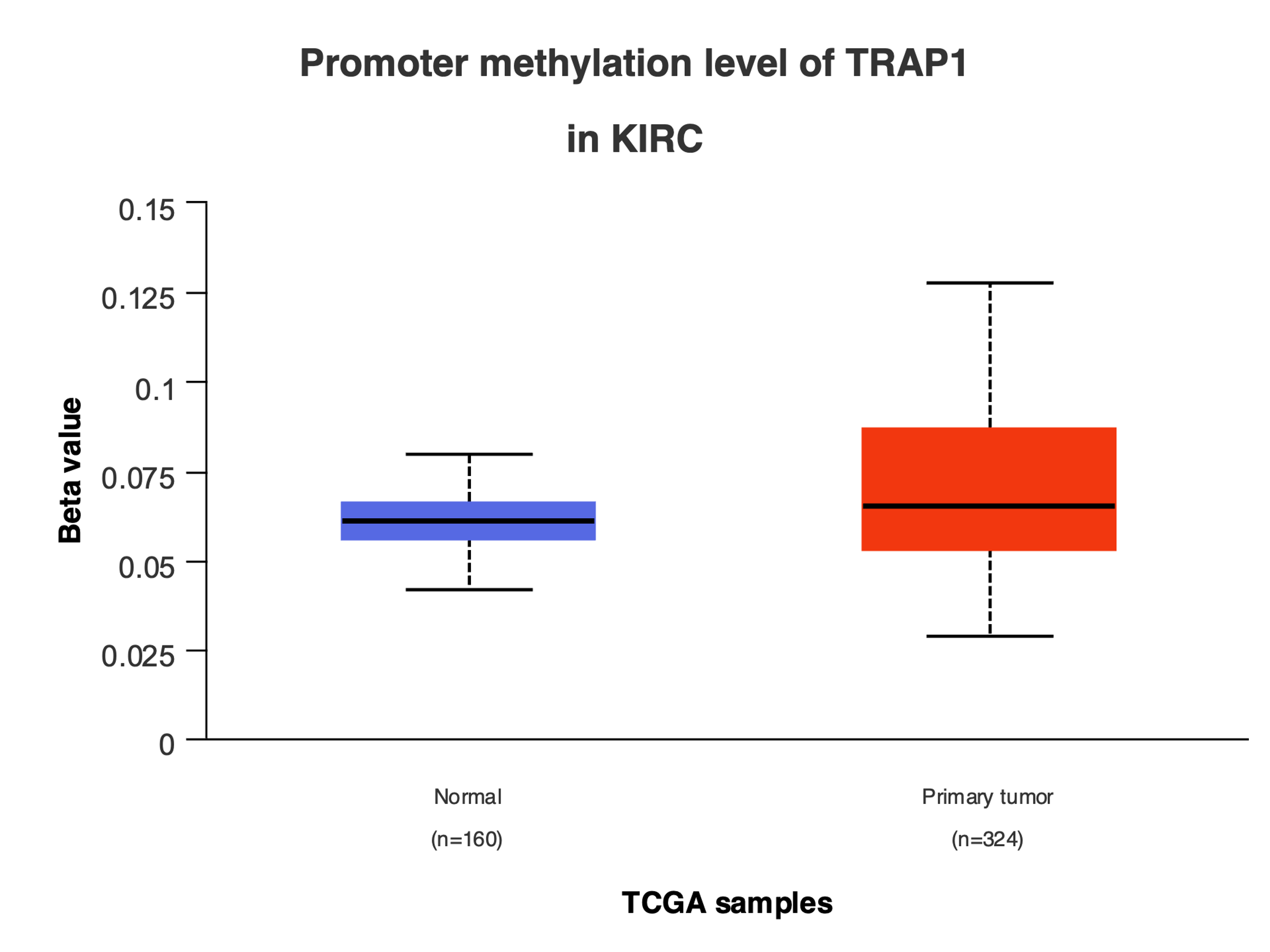


p=2.277e-11


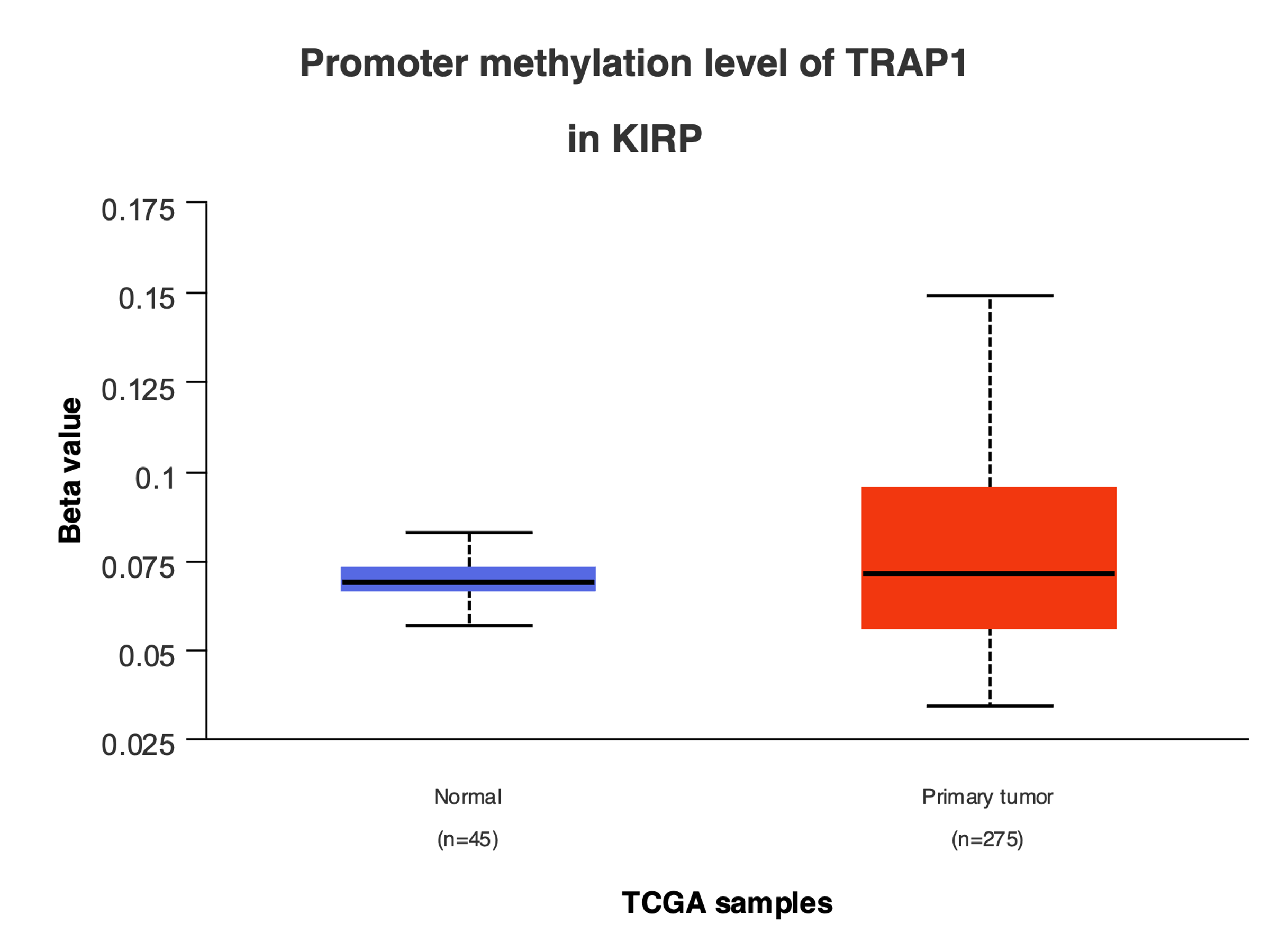


p=7.174e-04


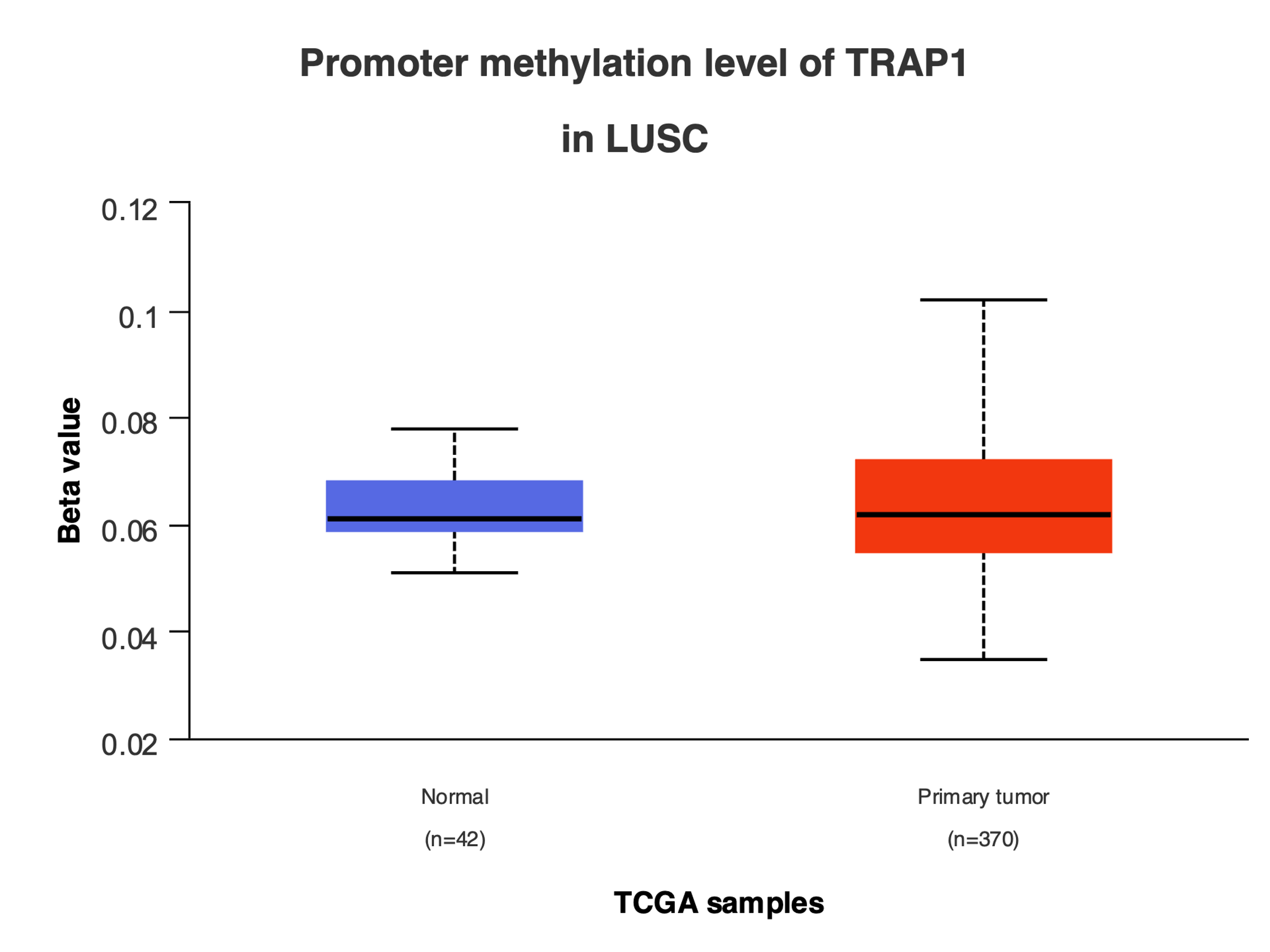


p=9.738e-03


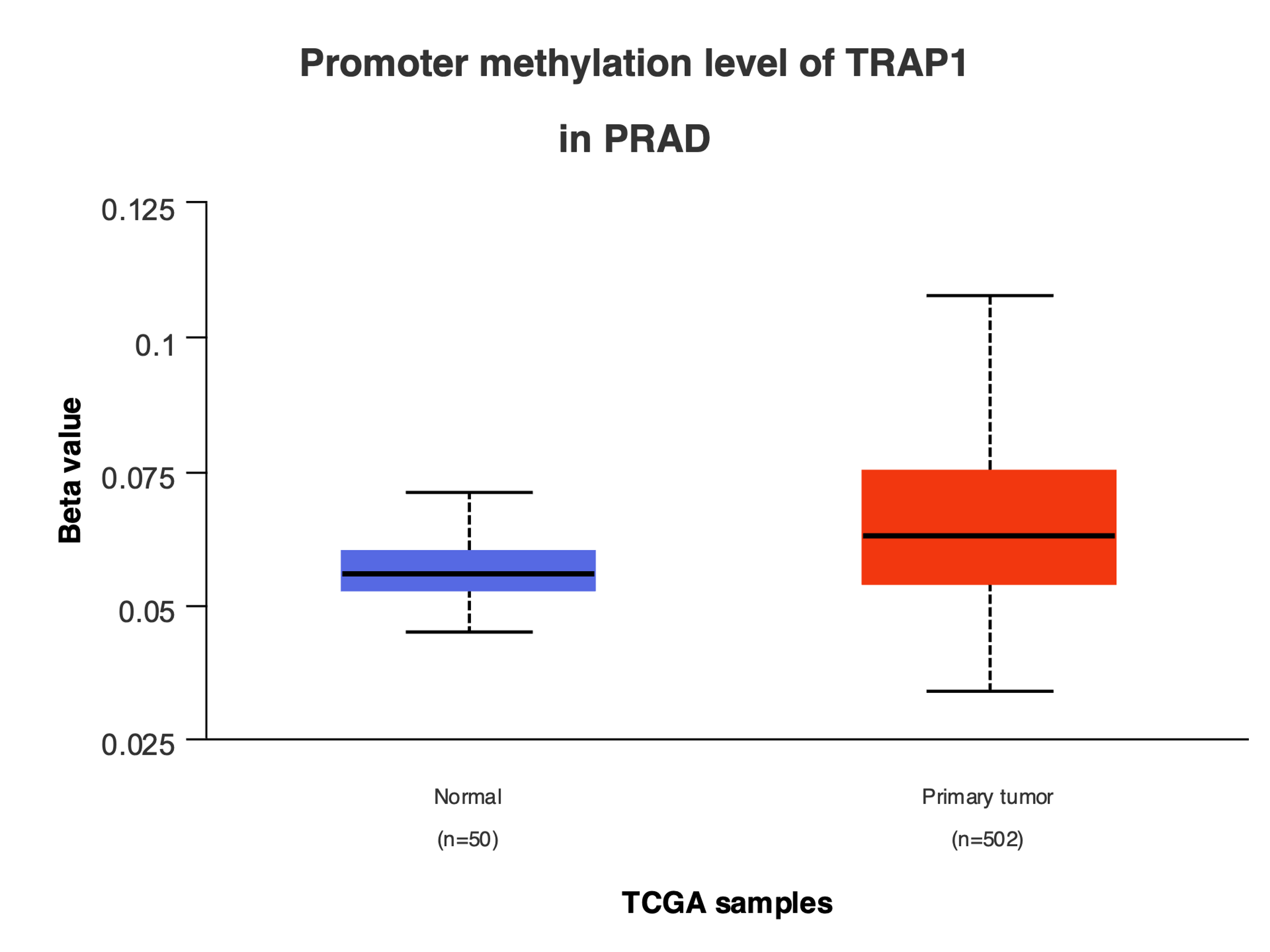


p=1.988e-09


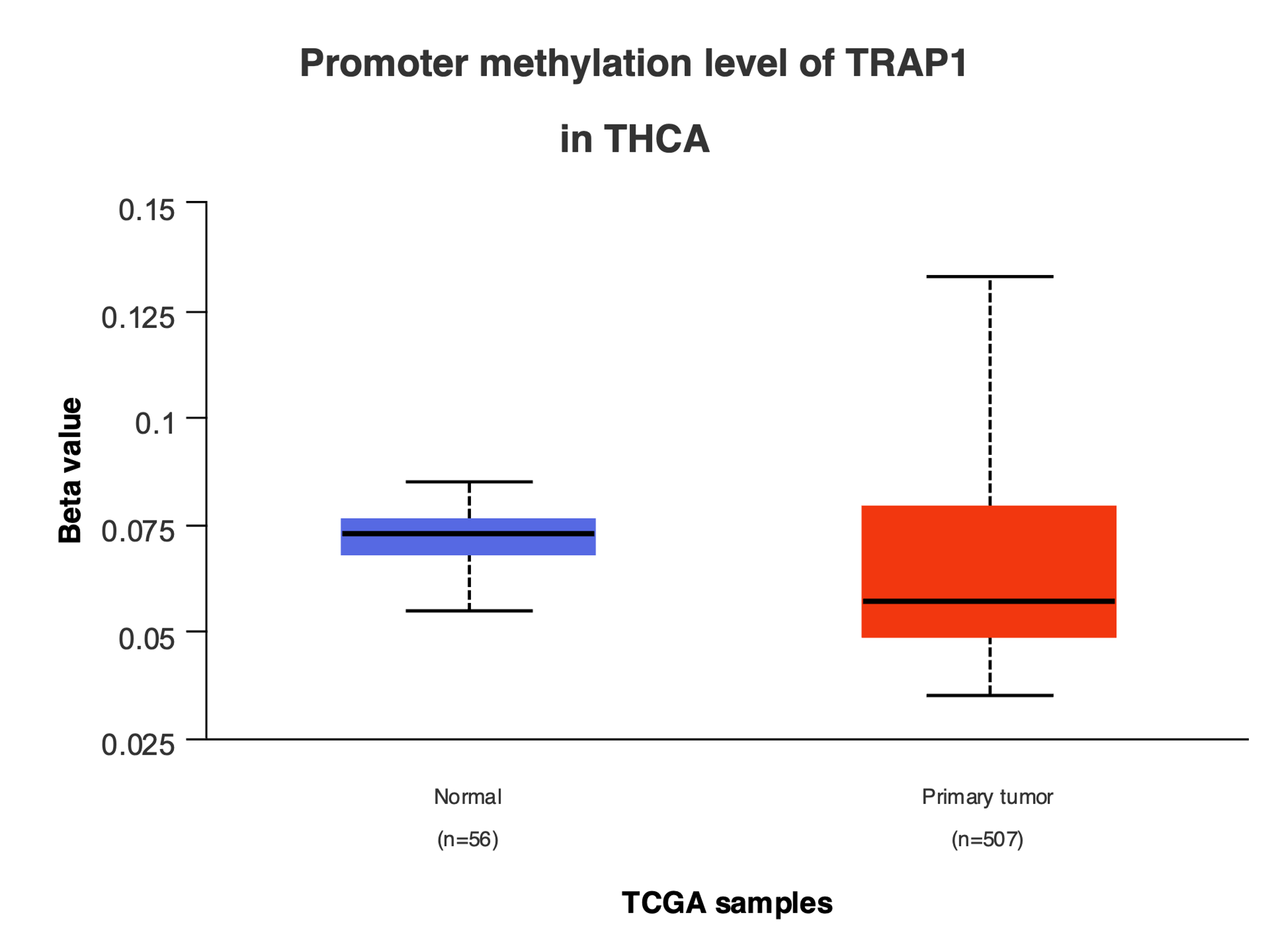


p=1.242e-02
