# Supplementary figures and images for: Comprehensive pan-cancer analysis of TRAP1 and its experimental validation in hepatocellular carcinoma
Source: Discov Oncol. 2025 Dec 20;17:152. doi: 10.1007/s12672-025-04238-9 (PMC12835488; doi:10.1007/s12672-025-04238-9)

TRAP1's connection to immune infiltration in 34 cancer types.
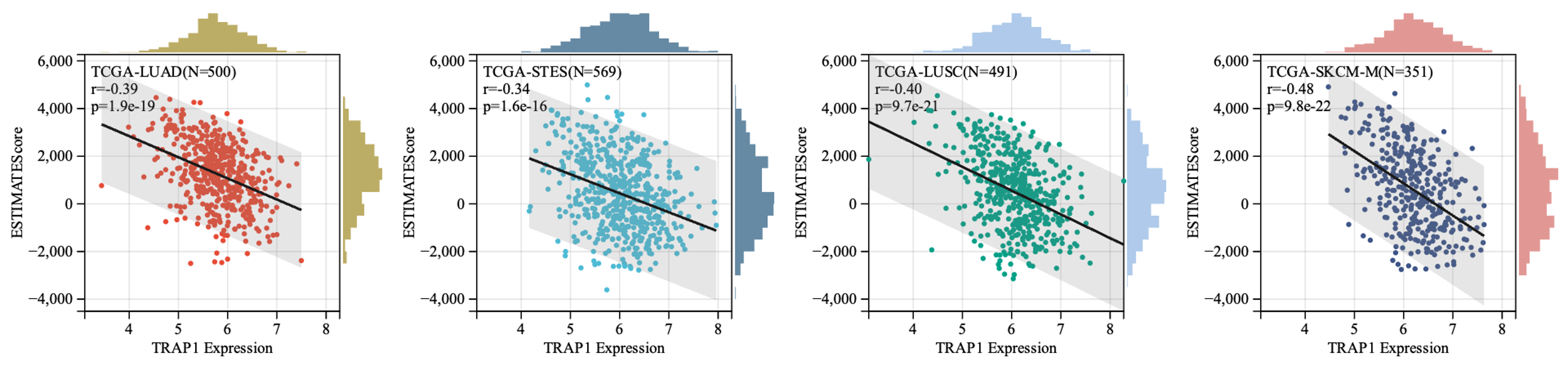

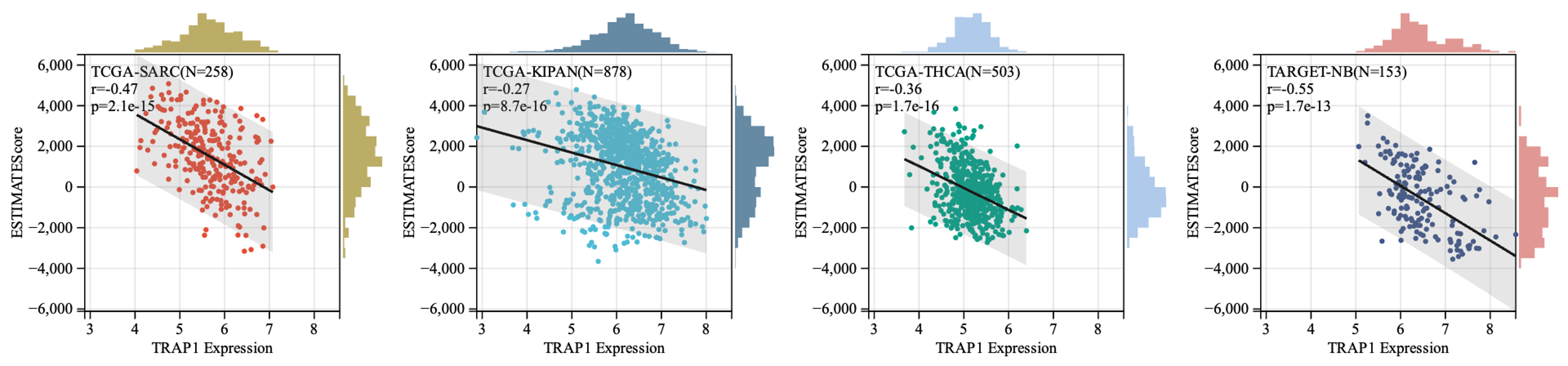

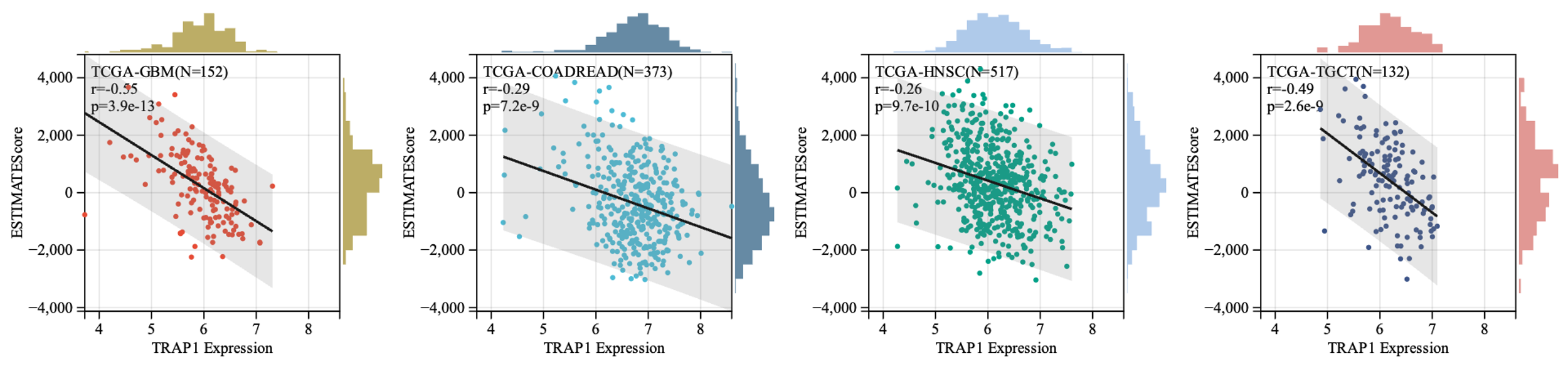

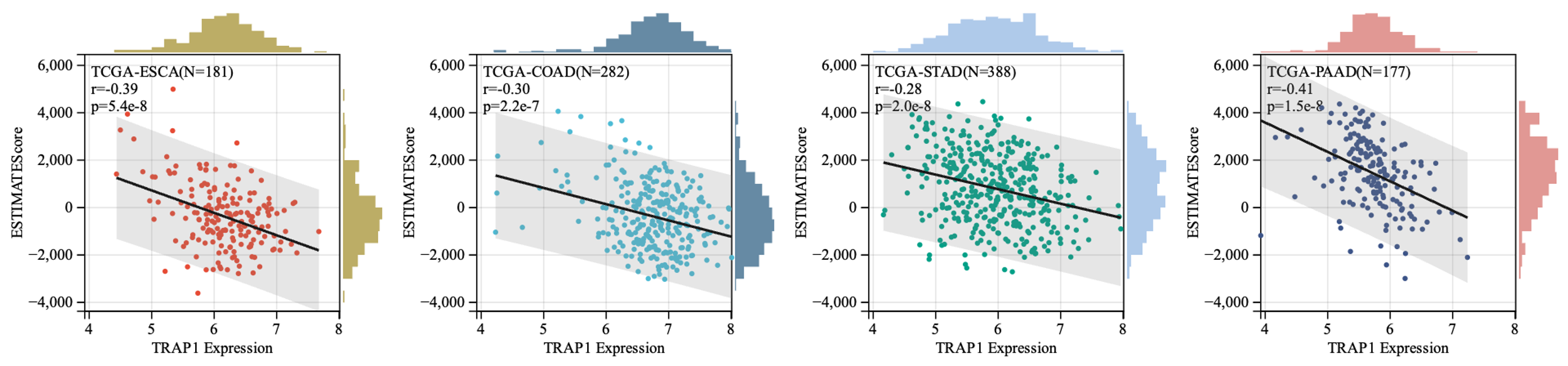

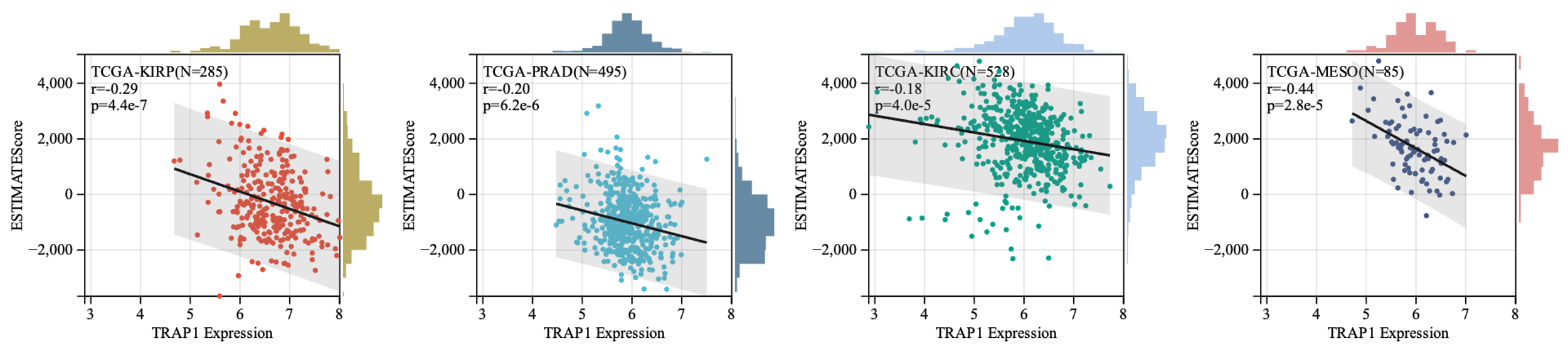

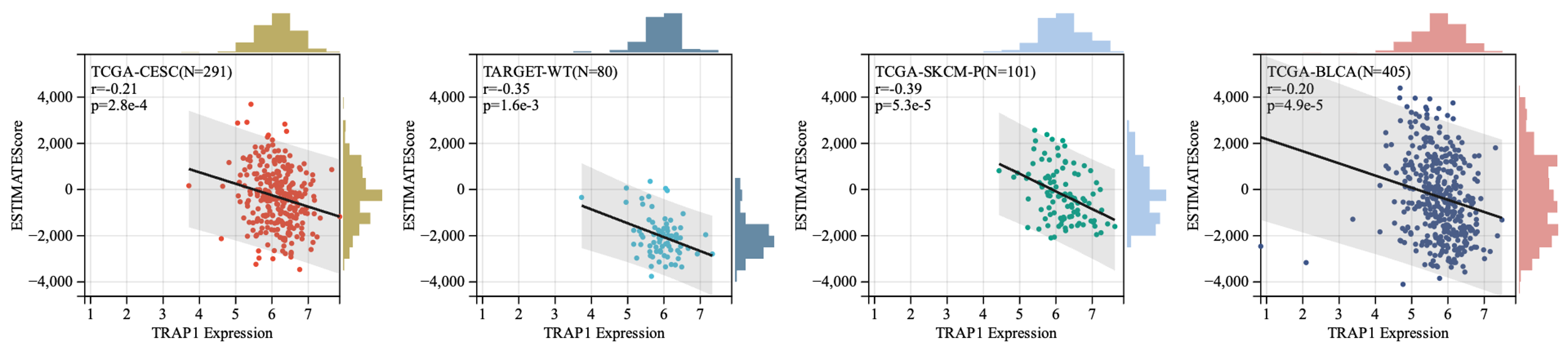

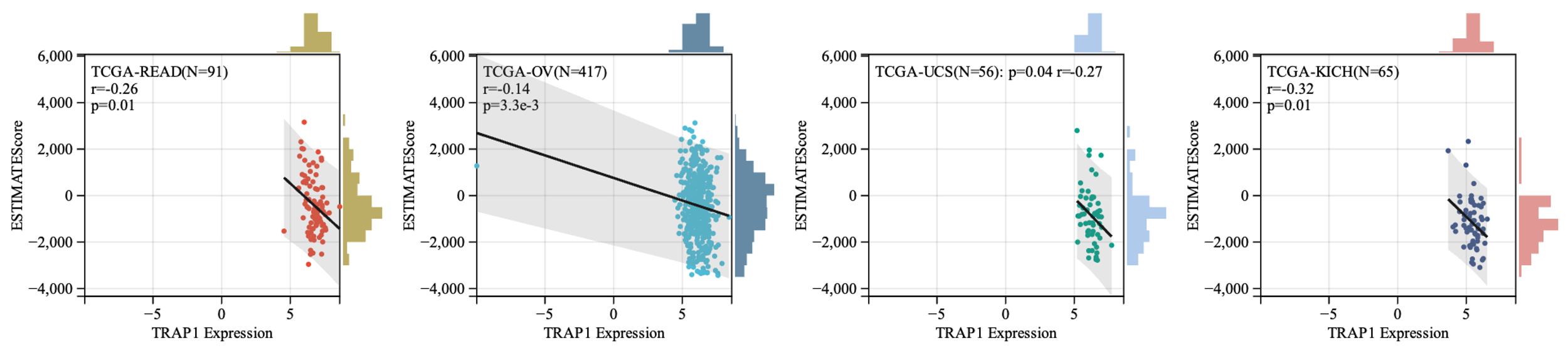

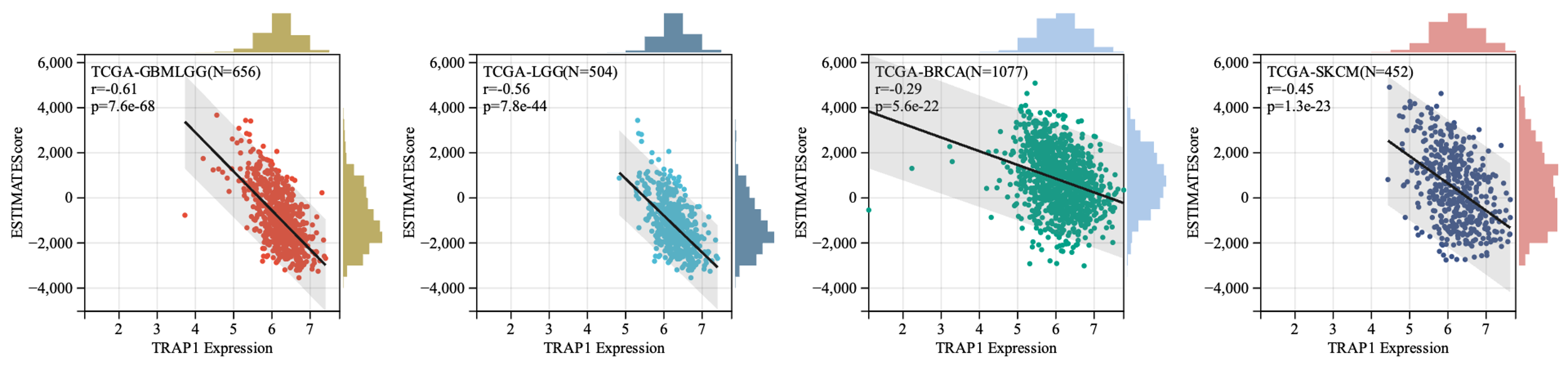

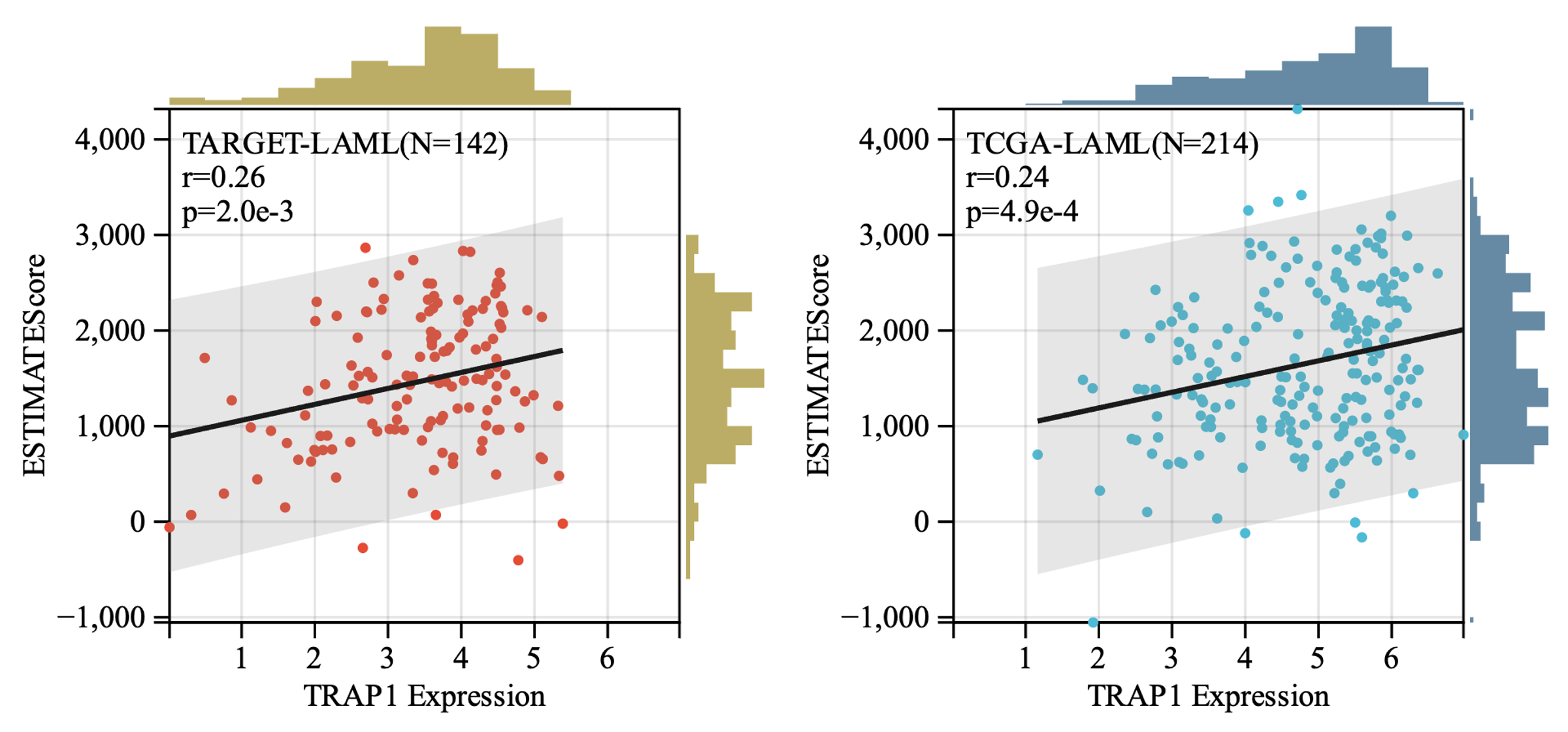

Supplement: Supplementary file 5 — Supplementary Material 5. [file 12672_2025_4238_MOESM5_ESM.docx]

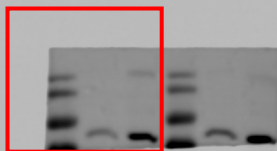

shTRAP1 Control

Supplement: Supplementary file 7 — Supplementary Material 7. [file 12672_2025_4238_MOESM7_ESM.pdf]

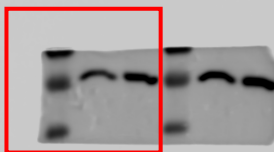

shTRAP1 Control

Supplement: Supplementary file 8 — Supplementary Material 8. [file 12672_2025_4238_MOESM8_ESM.pdf]

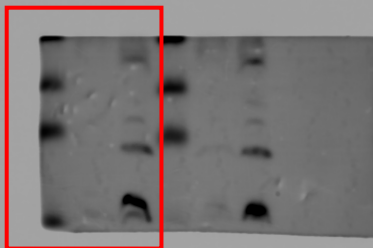

Control shTRAP1

Supplement: Supplementary file 9 — Supplementary Material 9. [file 12672_2025_4238_MOESM9_ESM.pdf]

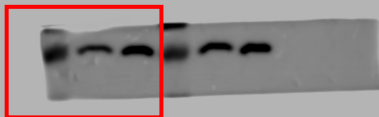

Control shTRAP1

Supplement: Supplementary file 10 — Supplementary Material 10. [file 12672_2025_4238_MOESM10_ESM.pdf]

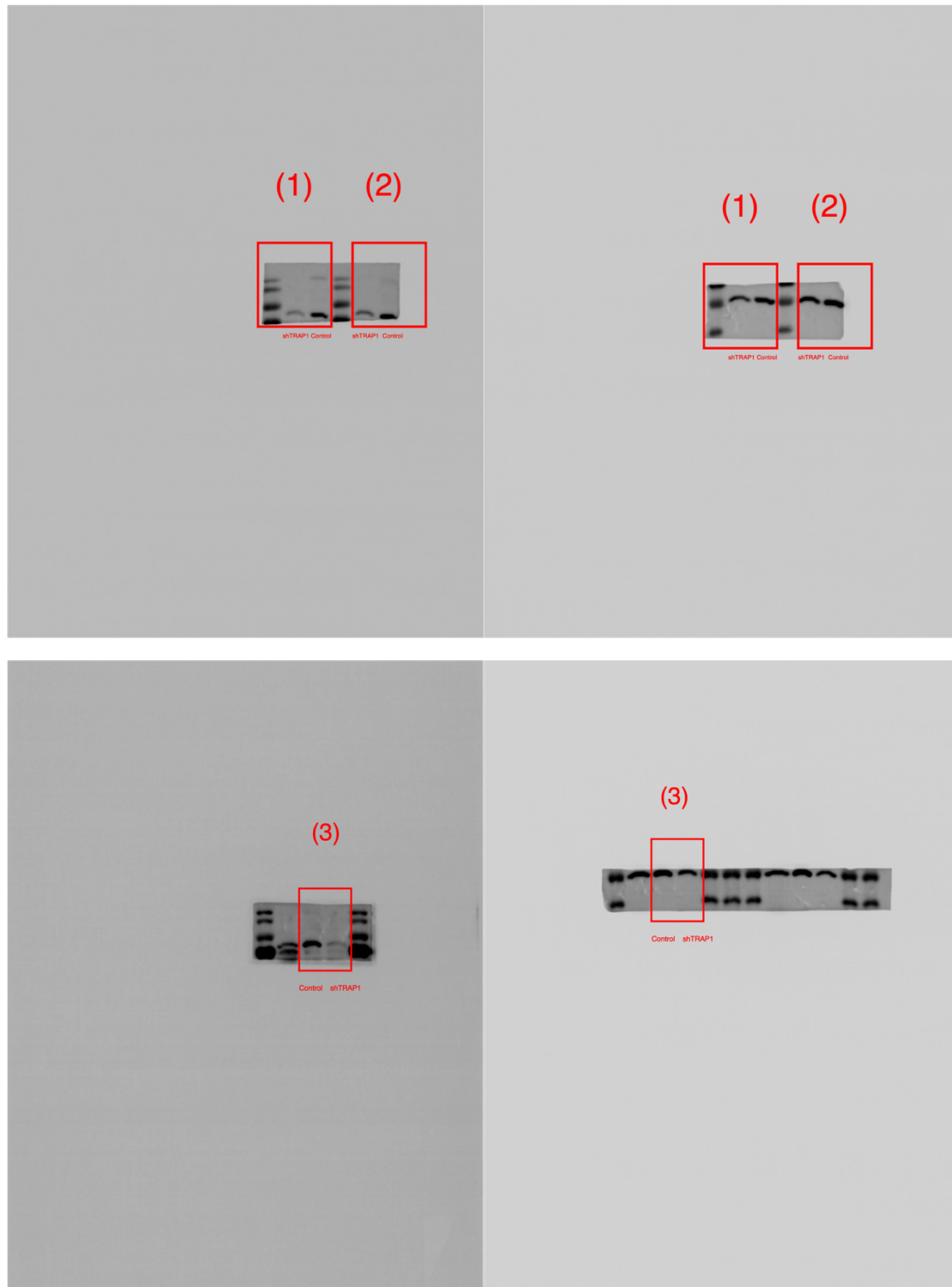

*Fig.8B* TRAP1

$\alpha$ -tubulin

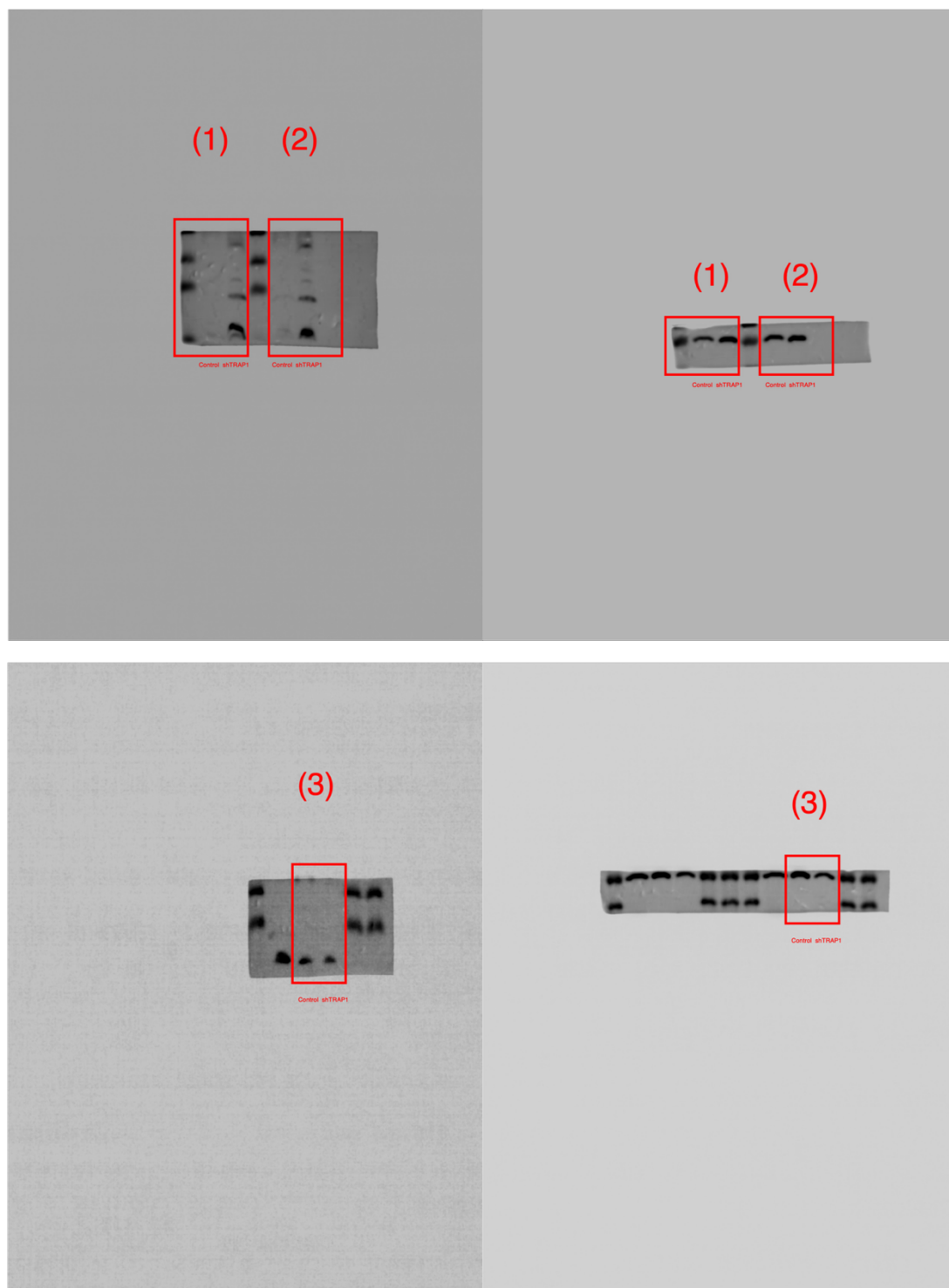

*Fig.8C* caspase-3

α-tubulin

Supplement: Supplementary file 11 — Supplementary Material 11. [file 12672_2025_4238_MOESM11_ESM.pdf]
